# Oxovanadium(v)-catalyzed amination of carbon dioxide under ambient pressure for the synthesis of ureas†

**DOI:** 10.1039/d1ra04125h

**Published:** 2021-08-09

**Authors:** Toshiyuki Moriuchi, Takashi Sakuramoto, Takanari Matsutani, Ryota Kawai, Yosuke Donaka, Mamoru Tobisu, Toshikazu Hirao

**Affiliations:** Department of Applied Chemistry, Graduate School of Engineering, Osaka University Yamada-oka, Suita Osaka 565-0871 Japan moriuchi@sci.osaka-cu.ac.jp; Division of Molecular Materials Science, Graduate School of Science, Osaka City University 3-3-138 Sugimoto, Sumiyoshi-ku Osaka 558-8585 Japan

## Abstract

Carbon dioxide is regarded as a reliable C1 building block in organic synthesis because of the nontoxic, abundant, and economical characteristics of carbon dioxide. In this manuscript, a commercially available oxovanadium(v) compound was demonstrated to serve as an efficient catalyst for the catalytic amination of carbon dioxide under ambient pressure in the synthesis of ureas. The catalytic transformation of chiral amines into the corresponding chiral ureas without loss of chirality was also performed. Furthermore, a gram-scale catalytic urea synthesis under ambient pressure was successfully achieved to validate the scalability of this catalytic activation of carbon dioxide.

## Abstract 2

Catalytic activation of carbon dioxide as a C1 building block under ambient pressure was performed by using a commercially available oxovanadium(v) catalyst for the synthesis of ureas.

## Graphical abstract

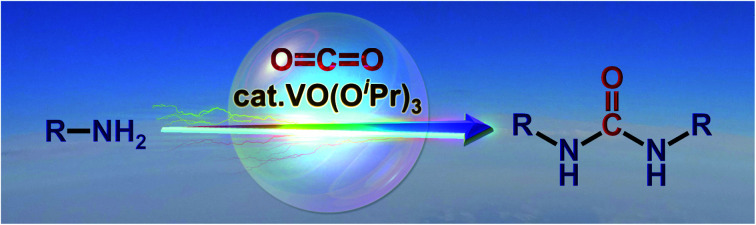

## Introduction

**Scheme 1:**
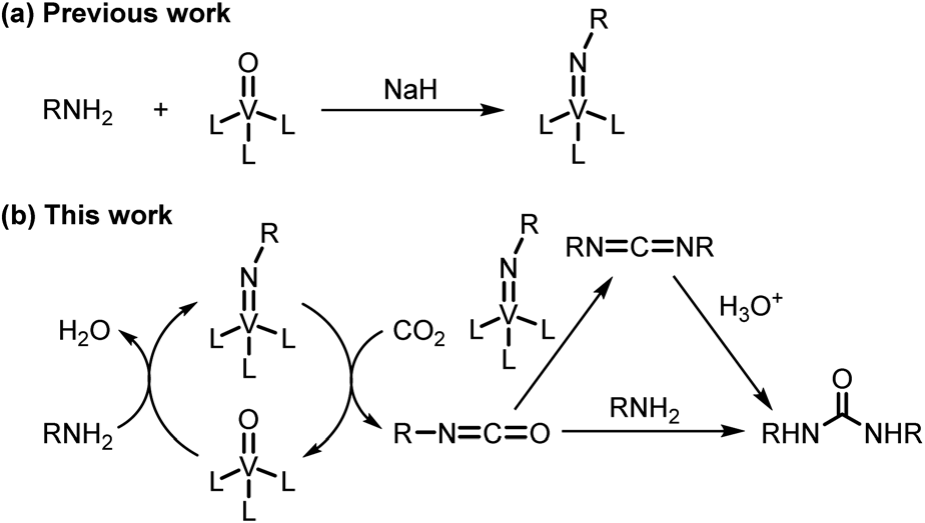
(a) Previous work: one-step synthesis of imidovanadium(v) compounds. (b) This work: oxovanadium(v)-catalyzed amination of carbon dioxide for the synthesis of ureas.

Efficient utilization of carbon dioxide is considered to be essential for the future sustainable society.^1^ Especially, the employment of carbon dioxide as a C1 building block permits the synthesis of valuable compounds such as ureas, which are widely used as pesticides, herbicides, and raw materials of resin. Some catalytic systems using carbon dioxide for the synthesis of ureas have been reported,^2^ but generally require high carbon dioxide pressure and high temperature. The catalytic system under ambient pressure is limited to CsOH/ionic liquid^3^ and TBA_2_[WO_4_]^4^ systems. These systems, however, use expensive ionic liquid as a solvent or lack versatility of substrates. Also, the synthesis of a cyclic urea by the direct reaction of carbon dioxide and diamine without the use of a catalyst requires high carbon dioxide pressure.^5^ Reaction of carbon dioxide with imidometal compounds provides a route to the isocyanates.^6^ Even though stoichiometric reaction of carbon dioxide with imidotitanium compounds at ambient temperature and under ambient pressure has been conducted to afford ureas,^6*b*^ catalytic reactions with *in situ* generated imidometal compounds have not been achieved. We have already developed the one-step synthetic protocol of imidovanadium(v) compounds from amines and oxovanadium(v) compounds (Scheme 1a).^7^ Application of *in situ* generated imidovanadium(v) compounds from amines for transformation reactions of nitrogen functional groups has not been performed although there are catalytic systems using isocyanates^8^ or azobenzenes^9^ instead of amines. From these points of view, we embarked upon the development of a catalytic carbon dioxide activation system for the synthesis of ureas by utilizing *in situ* generated imidovanadium(v) compounds from amines and a commercially available oxovanadium(v) compound (Scheme 1b).

## Results and discussion

For the first approach, we began our investigation by screening whether oxovanadium(v) compounds could serve as a catalyst in the catalytic amination of carbon dioxide under ambient pressure for the synthesis of ureas. The reaction of 2-phenylethylamine (1a) with carbon dioxide (balloon) in the presence of VO(O^*i*^Pr)_3_ as a catalyst, *N*,*N*-diisopropylethylamine (^*i*^Pr_2_EtN) as a base and molecular sieve (MS3A) as a dehydrating reagent in *N*,*N*-dimethylacetamide (DMA) was found to afford the corresponding urea 2a in 45% yield (Table 1, entry 1). Encouraged by this initial result, the effect of oxovanadium(v) compounds was examined. The reaction in the absence of VO(O^*i*^Pr)_3_ resulted in a lower yield (entry 2), revealing the efficiency of VO(O^*i*^Pr)_3_ catalyst for the amination of carbon dioxide in the synthesis of the urea 2a. The reactivity of oxovanadium(v) compounds was found to be changed by the ligands binding to the vanadium metal center. The Lewis acidity of oxovanadium(v) compounds is known to be increased by replacing the alkoxy ligand with the chloride ligand.^10^ The yield of the urea 2a decreased with increasing Lewis acidity of oxovanadium(v) compounds (entries 3–5). VO(TEA)^11^ and V_2_O_5_ displayed no promising results (entries 6–7). When (NH_4_)_2_MoO_4_ was used instead of VO(O^*i*^Pr)_3_, the urea 2a was obtained in a lower yield (entry 8). The oxo-metal compounds including TiO_2_, WO_3_, FeO, Fe_2_O_3_, NbO_2_ and Nb_2_O_5_ were not effective as catalysts (entries 9–14).

**Table 1:** Metal-catalyzed urea formation from 2-phenylethylamine (1a) and carbon dioxide^a^

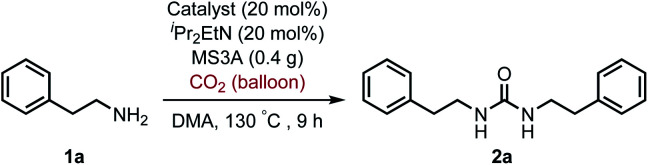		
Entry	Catalyst	NMR yield^b^ (%)
1	VO(O^*i*^Pr)_3_	45
2	—	5
3	VO(Et)_3_^c^	25
4	VO(OEt)Cl_2_	22
5	VOCl_3_	17
6	VO(TEA)^d^	3
7	V_2_O_5_	6
8	(NH_4_)_2_MoO_4_	21
9	TiO_2_	5
10	WO_3_	4
11	FeO	9
12	Fe_2_O_3_	9
13	NbO_2_	6
14	Nb_2_O_5_	7

a Reaction conditions: 1a (0.60 mmol), catalyst (20 mol%), ^*i*^Pr_2_EtN (20 mol%) and MS3A (0.4 g) in DMA (2.0 mL) under carbon dioxide (ballon) at 130 °C for 9 h.

b NMR (%) = [product (mmol) × 2/substrate (mmol)] × 100.

c 15 mol%, 6 h.

d
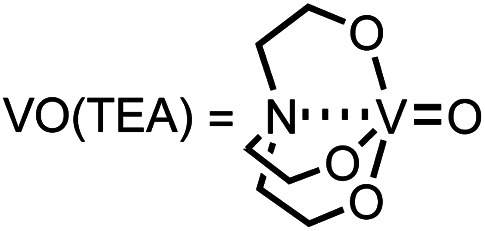

To improve the transformation outcome, the effect of various reaction parameters was examined (Table 2). An increase in the amount of MS3A significantly improved the yield of the urea 2a (entries 1–4). The reaction was further optimized by changing the concentration. Decreasing substrate concentration increased the yield to 91% (entry 5) although higher substrate concentration resulted in a lower yield (entry 6). The amount of catalyst as well as substrate concentration is important in this catalytic system (entries 7–9). Eventually, the amount of the catalyst loading could be reduced to 8 mol% to afford the desired urea 2a in 93% yield with a longer reaction time (entry 9). The effect of ^*i*^Pr_2_EtN was examined. A decrease in the amount of ^*i*^Pr_2_EtN resulted in a lower yield (entries 10–11). Even with a reaction time of 5 h, the urea 2a was obtained in 81% yield (entry 12). The choice of solvent is also an important factor in the efficient catalytic activity (Table S1, ESI†). *N*,*N*-Dimethylformamide (DMF) and 1-methyl-2-pyrrolidone (NMP) as well as dimethyl sulfoxide (DMSO) were found to be less effective solvents in this catalytic transformation under the reaction conditions of entry 12 (DMF, 64%; NMP, 56%; DMSO, 40%). The utilization of mesitylene and octane led to low yields of 17% and 13%, respectively. The efficiency of organic dehydrating reagents was examined. While *N*,*O*-bis(trimethylsilyl)acetamide showed no promising result (entry 13), the use of dimethylethylsilylimidazole instead of MS3A displayed similar efficiency to give the urea 2a in 81% yield (entry 14).

**Table 2:** Oxovanadium(v)-catalyzed urea formation from 2-phenylethylamine (1a) and carbon dioxide^a^

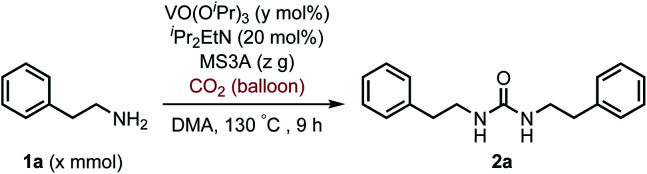				
Entry	1a (*x* mmol)	VO(O^*i*^Pr)_3_ (*y* mol%)	MS3A (*z* g)	NMR yield^b^ (%)
1	0.60	20	0.4	45
2	0.60	20	1.2	69
3	0.60	20	2.0	81
4	0.60	20	3.0	81
5	0.30	20	2.0	91
6	1.20	20	2.0	79
7	0.30	15	2.0	84
8	0.30	8	2.0	73
9^c^	0.15	8	2.0	93
10^c^^,^^d^	0.15	8	2.0	85
11^c^^,^^e^	0.15	8	2.0	84
12^f^	0.15	8	2.0	81
13^f^^,^^g^	0.15	8	2.0	26
14^f^^,^^h^	0.15	8	2.0	81
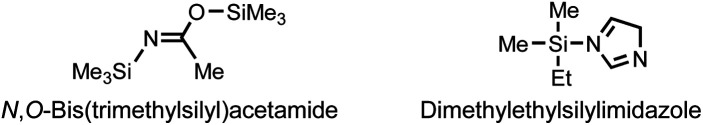				

a Reaction conditions: 1a (*x* mmol), VO(O^*i*^Pr)_3_ (*y* mol%), ^*i*^Pr_2_EtN (20 mol%) and MS3A (*z* g) in DMA (2.0 mL) under carbon dioxide (balloon) at 130 °C for 9 h.

b NMR yield (%) = [product (mmol) × 2/substrate (mmol)] × 100.

c For 12 h.

d With ^*i*^Pr_2_EtN (10 mol%).

e Without ^*i*^Pr_2_EtN.

f For 5 h.

g *N*,*O*-Bis(trimethylsilyl)acetamide (500 mol%) was used instead of MS3A.

h Dimethylethylsilylimidazole (500 mol%) was used instead of MS3A.

To explore the validity of this VO(O^*i*^Pr)_3_-catalyzed amination system, the substrate scope of amines in the synthesis of ureas was examined under the optimized reaction conditions (Table 3). In the case of primary aliphatic amines, the catalytic reaction proceeded well to afford the corresponding ureas 2b–p in good yields (entries 1–15). For example, this catalytic system could be applied to 2-(4-bromophenyl)ethylamine (1b), in which the obtained product can be utilized for further transformation using Br group (entry 1). Chiral amine, (*S*)-(−)-1-phenylethylamine (1i), was converted into the corresponding chiral urea 2i with retention of chirality as determined by chiral HPLC analysis (entry 8, ESI†). The tetrahydrofuran moiety was tolerated (entry 12). The catalytic transformation of an aromatic amine, *p*-anisidine (1q), resulted in a moderate yield of 37% (entry 16). Use of a secondary amine, di-*n*-butylamine (1r), which cannot be transformed to the imidovanadium(v) compound in this catalytic system, did not lead to the urea formation (entry 17).

**Table 3:** Substrate scope of amines in the catalytic synthesis of ureas^a^

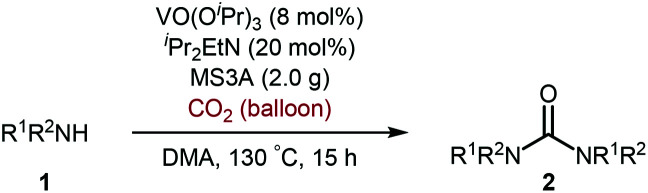					
Entry	Product	NMR yield^b^ (%)	Entry	Product	NMR yield^b^ (%)
1	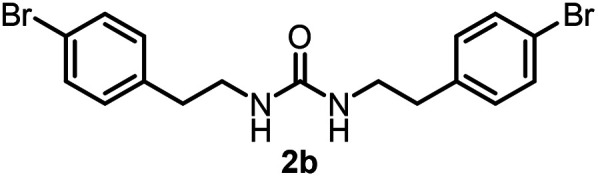	85	10	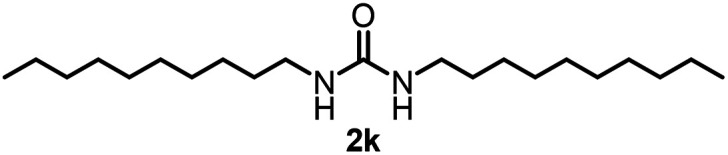	88
2	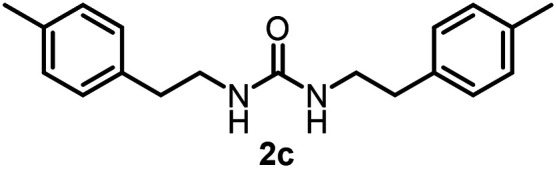	80	11	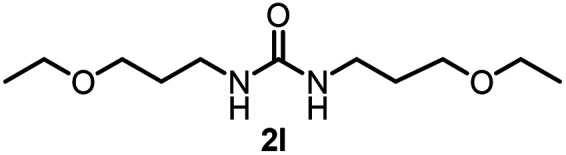	75
3	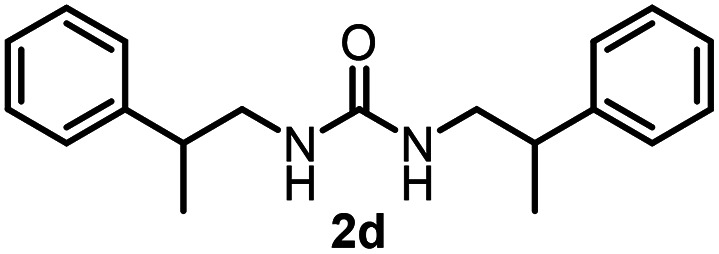	84	12	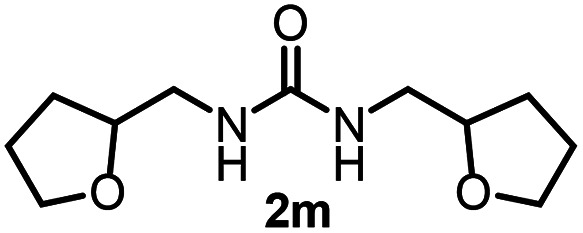	77
4	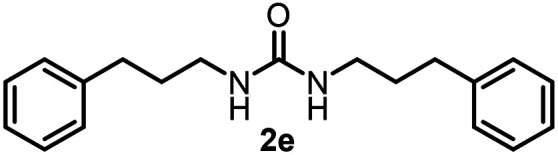	82	13	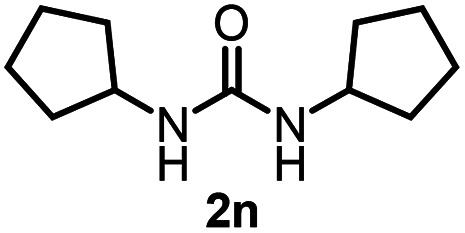	60
5	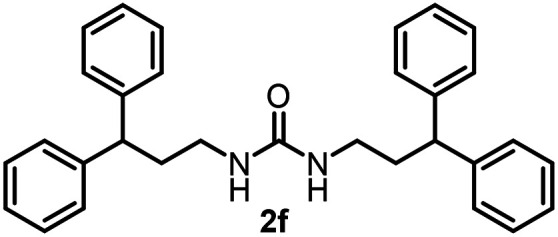	71	14	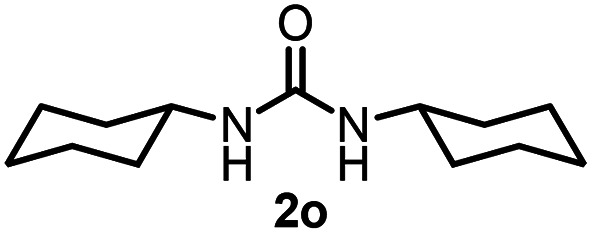	81
6	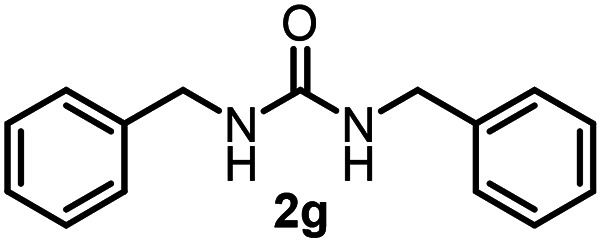	89	15	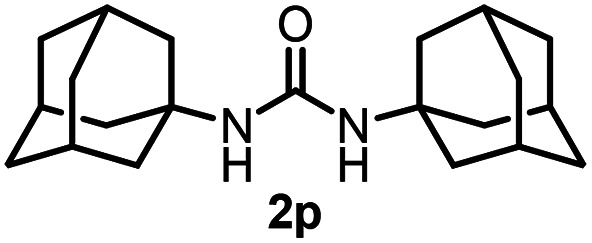	78^c^
7	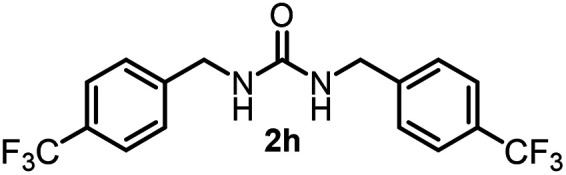	60	16	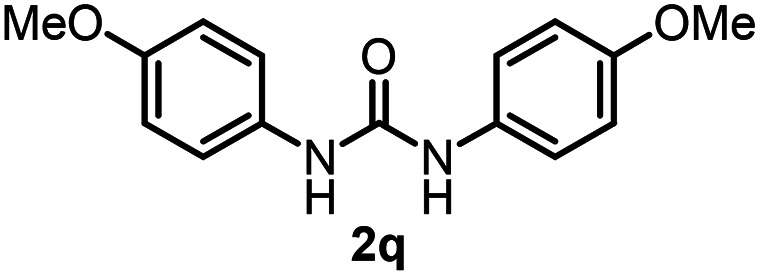	37^d^
8	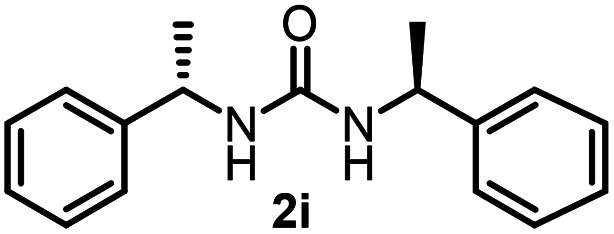	60	17	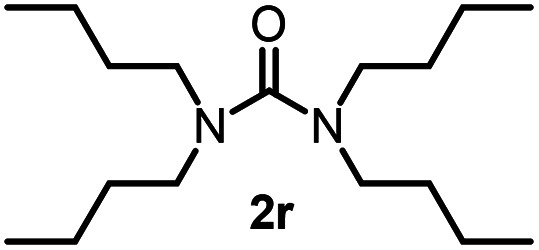	0
9	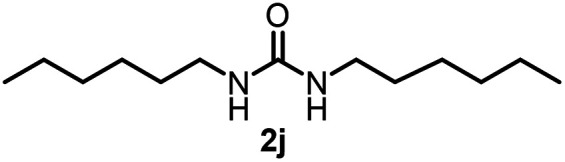	89			

a Reaction conditions: substrate 1 (0.15 mmol), VO(O^*i*^Pr)_3_ (8 mol%), ^*i*^Pr_2_EtN (20 mol%) and MS3A (2.0 g) in DMA (2.0 mL) under carbon dioxide (balloon) at 130 °C for 15 h.

b NMR yield (%) = [product (mmol) × 2/substrate (mmol)] × 100.

c Reaction time was 24 h.

d 15 mol% VO(O^*i*^Pr)_3_ was used.

**Scheme 2:**
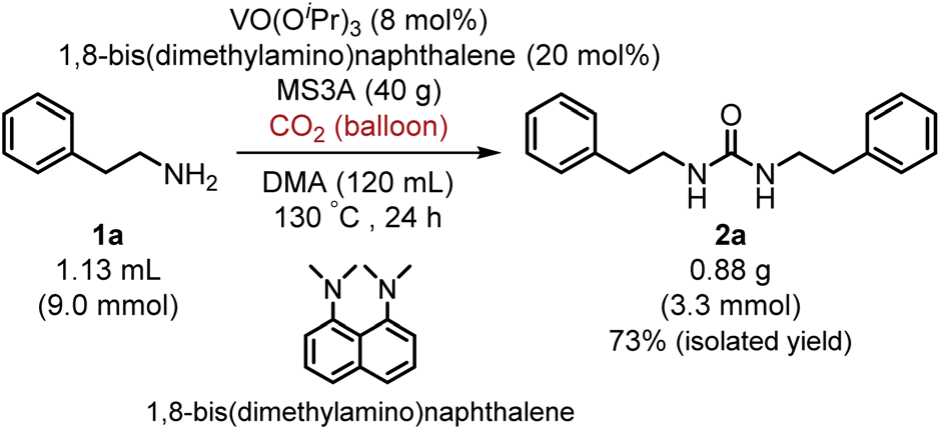
Gram-scale catalytic reaction of 2-phenylethylamine (1a) with carbon dioxide (balloon) by using VO(O^*i*^Pr)_3_ catalyst.

Moreover, it is worth mentioning that gram-scale catalytic reaction was successfully performed with 2-phenylethylamine (1a) by using 1,8-bis(dimethylamino)naphthalene instead of ^*i*^Pr_2_EtN to provide the urea 2a in 73% isolated yield (Scheme 2).

**Scheme 3:**
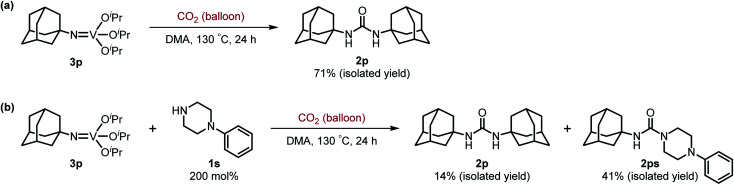
(a) Reaction of the imidovanadium(v) compound 3p with carbon dioxide (balloon). (b) Reaction of the imidovanadium(v) compound 3p with carbon dioxide (balloon) in the presence of 1-phenylpiperazine (1s).

**Scheme 4:**
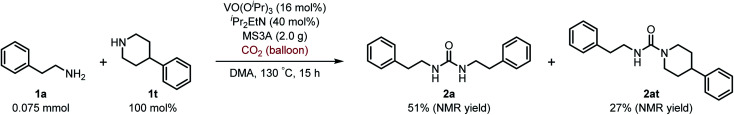
Reaction of 2-phenylethylamine (1a) with carbon dioxide (balloon) in the presence of 4-phenylpiperidine (1t) under the catalytic reaction conditions.

Next, we tackled a challenge to elucidate the reaction mechanism. To gain insight into the reactivity of the imidovanadium(v) compound with carbon dioxide, the imidovanadium(v) compound 3p, which was obtained by the reaction of 1-adamantylamine with VO(O^*i*^Pr)_3_ under atmospheric nitrogen, was heated in DMA under carbon dioxide (balloon) at 130 °C (Scheme 3a). The urea 2p was found to be obtained in 71% isolated yield after workup with 1 M aqueous HCl, indicating the formation of the carbodiimide which is probably produced in the reaction of the imidovanadium(v) compound 3p with the generated isocyanate.^8^ The difference in the reactivity of amines was applied to the detection of isocyanate formation. The reaction of the imidovanadium(v) compound 3p with carbon dioxide in the presence of a secondary amine, 1-phenylpiperazine (1s), which does not form the imidovanadium(v) compound, was carried out (Scheme 3b). In this reaction, not only the symmetric urea 2p (14% isolated yield) but also the unsymmetric urea 2ps (41% isolated yield) was produced, wherein the symmetric urea derived from 1s was not obtained. This result suggests that 1-adamantyl isocyanate might be generated to provide the unsymmetric urea 2ps. Furthermore, the reaction of 2-phenylethylamine (1a) with carbon dioxide in the presence of a secondary amine, 4-phenylpiperidine (1t), under the catalytic reaction conditions (Scheme 4) was performed to provide the unsymmetric urea 2at (27% NMR yield) with formation of the symmetric urea 2a (51% NMR yield). Based on the thus-obtained preliminary mechanistic studies, we propose the catalytic cycle outlined in Scheme 1b. The amine reacts with VO(O^*i*^Pr)_3_ to form the imidovanadium(v) compound, which can then react with carbon dioxide to generate the corresponding isocyanate. The desired urea product is produced either by the reaction of the generated isocyanate with the starting amine or through the formation of the carbodiimide.

## Conclusions

Catalytic activation of carbon dioxide as a C1 building block under ambient pressure for the synthesis of ureas was achieved by using a commercially available oxovanadium(v) compound. This catalytic system efficiently transforms various primary amines into the ureas. Our method is applicable to the synthesis of chiral urea without loss of chirality. Furthermore, a gram-scale catalytic reaction was also successfully performed to validate the scalability of this catalytic activation of carbon dioxide. Further studies aiming to achieve other types of catalytic activation of carbon dioxide by this strategy are currently under investigation.

## Note added after first publication

This article replaces the version published on 9th August 2021, which contained errors in Table 1.

## Conflicts of interest

There are no conflicts to declare.

## Supplementary Material

RA-011-D1RA04125H-s001
