# Correction: Oxovanadium(v)-catalyzed amination of carbon dioxide under ambient pressure for the synthesis of ureas

**DOI:** 10.1039/d1ra90159a

**Published:** 2021-10-18

**Authors:** Toshiyuki Moriuchi, Takashi Sakuramoto, Takanari Matsutani, Ryota Kawai, Yosuke Donaka, Mamoru Tobisu, Toshikazu Hirao

**Affiliations:** Department of Applied Chemistry, Graduate School of Engineering, Osaka University Yamada-oka, Suita Osaka 565-0871 Japan moriuchi@sci.osaka-cu.ac.jp; Division of Molecular Materials Science, Graduate School of Science, Osaka City University 3-3-138 Sugimoto, Sumiyoshi-ku Osaka 558-8585 Japan

## Abstract

Correction for ‘Oxovanadium(v)-catalyzed amination of carbon dioxide under ambient pressure for the synthesis of ureas’ by Toshiyuki Moriuchi *et al.*, *RSC Adv.*, 2021, **11**, 27121–27125. DOI: 10.1039/D1RA04125H.

The authors regret that there were two errors in the original version of the manuscript.

The third entry of Table 1, which originally read VO(Et)_3_, should be VO(OEt)_3_. The superscript reference to footnote *c* remains correct.

In addition, the structure of dimethylethylsilylimidazole in Table 2 in the original manuscript was incorrect. The correct structure is given here:
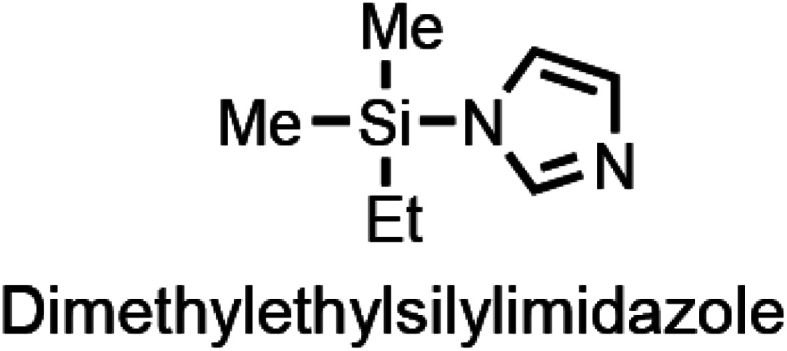


The Royal Society of Chemistry apologises for these errors and any consequent inconvenience to authors and readers.

## Supplementary Material

