# Supplementary material for: Oxovanadium(v)-catalyzed amination of carbon dioxide under ambient pressure for the synthesis of ureas
Source: RSC Adv. 2021 Aug 9;11(44):27121–5. doi: 10.1039/d1ra04125h (PMC9037642; doi:10.1039/d1ra04125h)
Supplement: RA-011-D1RA04125H-s001 [file RA-011-D1RA04125H-s001.pdf]

Electronic Supplementary Material (ESI) for RSC Advances.
This journal is © The Royal Society of Chemistry 2021

Supplementary Information

Oxovanadium(V)-Catalyzed Amination of Carbon Dioxide under Ambient Pressure for Synthesis of Ureas

Toshiyuki Moriuchi,*[ab] Takashi Sakuramoto,[a] Takanari Matsutani,[c] Ryota Kawai,[a] Yosuke Donaka,[c] Mamoru Tobisu [a] and Toshikazu Hirao [ad]

[a] *Department of Applied Chemistry, Graduate School of Engineering, Osaka University, Yamada-oka, Suita, Osaka 565-0871, Japan*

[b] Present address: *Division of Molecular Materials Science, Graduate School of Science, Osaka City University, 3-3-138 Sugimoto, Sumiyoshi-ku, Osaka 558-8585, Japan*

[c] *Division of Molecular Materials Science, Graduate School of Science, Osaka City University, 3-3-138 Sugimoto, Sumiyoshi-ku, Osaka 558-8585, Japan*

[d] Present address: *The Institute of Scientific and Industrial Research, Osaka University, Mihoga-oka, Ibaraki, Osaka 567-0047, Japan*

Contents

1. General methods **2**

2. General procedure for oxovanadium(V)-catalyzed urea formation **2**

3. Procedure for a gram-scale catalytic urea synthesis of 2a **2**

4. Reaction of 1-adamantylamine with $VO(O^iPr)_3$ **3**

5. Reaction of 3p with carbon dioxide **3**

6. Reaction of 3p with carbon dioxide in the presence of 1s **3**

7. Reaction of 1a with carbon dioxide in the presence of 1t **4**

8. Table S1 **5**

9. Spectral data of products and chiral HPLC charts **6**

10. References **26**

1. General Methods

All reagents and solvents were purchased from commercial sources and were further purified by the standard methods, if necessary. VO(TEA) [1] and the imidovanadium(V) compound **3p** [2] were prepared according to literature procedures. ^{1}H NMR and ^{13}C NMR spectra were recorded in $CDCl_3$ or DMSO-d_6 on a JEOL JNM-ECS 400 (400 MHz) spectrometer. Chemical shifts are given in δ (ppm) relative to the residual solvent signal as an internal standard. High resolution mass spectra (HRMS) were measured on a JEOL JMS-700 spectrometer. Column chromatography was performed with SiO_2 (Wakogel C-200). The analysis of the chiral urea product **2i** was carried out using HPLC (Chiralpak IA, hexane/$CHCl_3$/EtOH = 8:2:1, flow 0.5 mL/min, 254 nm).

2. General procedure for oxovanadium(V)-catalyzed urea formation

In a 10 mL two-necked flask, amine **1** (0.15 mmol), ^{i}Pr_2EtN (3.9 mg, 0.030 mmol), VO(O^{i}Pr)$_3$ (2.9 mg, 0.012 mmol), MS3A (2.0 g) and DMA (2.0 mL) were placed at a glove box filled with nitrogen. Next, nitrogen in the flask was replaced with CO_2. The mixture was stirred at 130 °C for 15 h, followed by the filtration with celite®, treatment with water, and extraction with EtOAc. The organic layer was dried over Na_2SO_4, filtrated, and evaporated. Triphenylmethane or 1,3,5-trimethoxybenzene was added as an internal standard, and ^{1}H NMR analysis was performed to determine an NMR yield. Spectral data of the products were identical with those of authentic samples.

3. Procedure for a gram-scale catalytic urea synthesis of 2a

In a 300 mL three-necked flask, 2-phenylethylamine (**1a**) (1.09 g, 9.0 mmol), 1,8-bis(dimethylamino)naphthalene (0.386 g, 1.8 mmol), VO(O^{i}Pr)$_3$ (0.176 g, 0.72 mmol), MS3A (40 g) and DMA (120 mL) were placed at a glove box filled with nitrogen. Next, nitrogen in the flask was replaced with CO_2. The mixture was stirred at 130 °C for 24 h, followed by the filtration with celite®, treatment with water, and extraction with EtOAc. The organic layer was dried over Na_2SO_4, filtrated, and evaporated. The residue was chromatographed on a silica gel column eluting with hexane and ethyl acetate (1/3, v/v) to give 0.88 g (73% yield) of *N*,*N'*-bis(2-phenylethyl)urea (**2a**).

4. Reaction of 1-adamantylamine with $VO(O^iPr)_3$

In a 20 mL two-necked flask, 1-adamantylamine (**1p**) (90.8 mg, 0.60 mmol), $^iPr_2EtN$ (77.5 mg, 0.60 mmol), $VO(O^iPr)_3$ (146.8 mg, 0.60 mmol), 1,3,5-trimethoxybenzene (50.5 mg, 0.30 mmol) as an internal standard, MS3A (2.0 g) and DMA (2.0 mL) were placed at a glove box filled with nitrogen. The mixture was stirred at 130 °C for 2 h. After the reaction, ^1H NMR analysis was performed to determine an NMR yield. The imidovanadium(V) compound **3p** was found to be formed in 22% NMR yield.

5. Reaction of 3p with carbon dioxide

In a 10 mL two-necked flask, the imidovanadium(V) compound **3p** (56.6 mg, 0.15 mmol) and DMA (2.0 mL) were placed at a glove box filled with nitrogen. Next, nitrogen in the flask was replaced with CO_2. The mixture was stirred at 130 °C for 24 h, followed by treatment with 1 M HCl solution and extraction with dichloromethane. The organic layer was dried over Na_2SO_4, filtrated, and evaporated. The residue was subjected to preparative TLC (ethyl acetate/dichloromethane = 1:40) to afford 17.6 mg (71% yield) of *N,N'*-bis(tricyclo[3.3.1.1^{3,7}]dec-1-yl)urea (**2p**).

6. Reaction of 3p with carbon dioxide in the presence of 1s

In a 10 mL two-necked flask, the imidovanadium(V) compound **3p** (56.6 mg, 0.15 mmol), 1-phenylpiperazine (**1s**) (48.2 mg, 0.30 mmol) and DMA (2.0 mL) were placed at a glove box filled with nitrogen. Next, nitrogen in the flask was replaced with CO_2. The mixture was stirred at 130 °C for 24 h, followed by treatment with 1 M HCl solution and extraction with dichloromethane. The organic layer was dried over Na_2SO_4, filtrated, and evaporated. The residue was subjected to preparative TLC (ethyl acetate/dichloromethane = 1:1) to give 3.5 mg (14% yield) of *N,N'*-bis(tricyclo[3.3.1.1^{3,7}]dec-1-yl)urea (**2p**) and 20.8 mg (41% yield) of 4-phenyl-*N*-tricyclo[3.3.1.1^{3,7}]dec-1-yl-1-piperazinecarboxamide (**2ps**).

7. Reaction of 1a with carbon dioxide in the presence of 1t

In a 10 mL two-necked flask, 2-phenylethylamine (**1a**) (9.1 mg, 0.075 mmol), 4-phenylpiperidine (**1t**) (12.1 mg, 0.075 mmol), $^{i}Pr_2EtN$ (3.9 mg, 0.030 mmol), $VO(O^{i}Pr)_3$ (2.9 mg, 0.012 mmol), MS3A (2.0 g) and DMA (2.0 mL) were placed at a glove box filled with nitrogen. Next, nitrogen in the flask was replaced with CO_2. The mixture was stirred at 130 °C for 15 h, followed by the filtration with celite®, treatment with water, and extraction with EtOAc. The organic layer was dried over Na_2SO_4, filtrated, and evaporated. 1,3,5-Trimethoxybenzene was added as an internal standard, and ^{1}H NMR analysis was performed to determine an NMR yield (*N*,*N'*-bis(2-phenylethyl)urea (**2a**) and 4-phenyl-*N*-(2-phenylethyl)-1-piperidinecarboxamide (**2at**) in 51% and 27% yields, respectively).

8. Table S1

Table S1 Oxovanadium(V)-catalyzed urea formation from 2-phenylethylamine and carbon dioxide.[a]

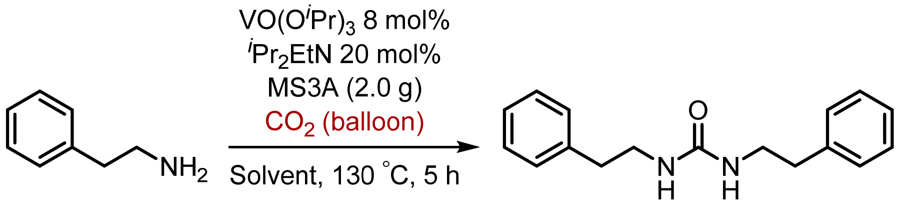

1a **2a**

Entry	Solvent	NMR yield (%) [b]
1	DMA	81
2	DMF	64
3	NMP	56
4	DMSO	40
5	mesitylene	17
6	octane	13

[a] Reaction conditions: **1a** (0.15 mmol), catalyst (8 mol%), $^{i}Pr_2EtN$ (20 mol%) and MS3A (2.0 g) in DMA (2.0 mL) under carbon dioxide (balloon) at 130 °C for 5 h. [b] NMR yield (%) = [product (mmol) x 2 / substrate (mmol)] x 100.

9. Spectral data of products and chiral HPLC charts

N,*N'*-Bis(2-phenylethyl)urea (**2a**) [CAS Registry No. 5467-84-5]: ^{1}H NMR (400 MHz, $CDCl_3$) δ 7.32-7.16 (m, 10H), 4.12 (br, 2H), 3.43-3.38 (m, 4H), 2.79 (t, J = 6.8 Hz, 4H); ^{13}C NMR (100 MHz, $CDCl_3$) 157.9, 139.3, 129.0, 128.8, 126.6, 41.8, 36.5 ppm; HRMS (FAB) *m/z* Calcd. for $C_{17}H_{21}N_2O$ ([M + H]$^{+}$), 269.1648; Found, 269.1652.

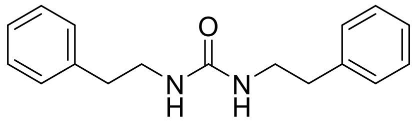

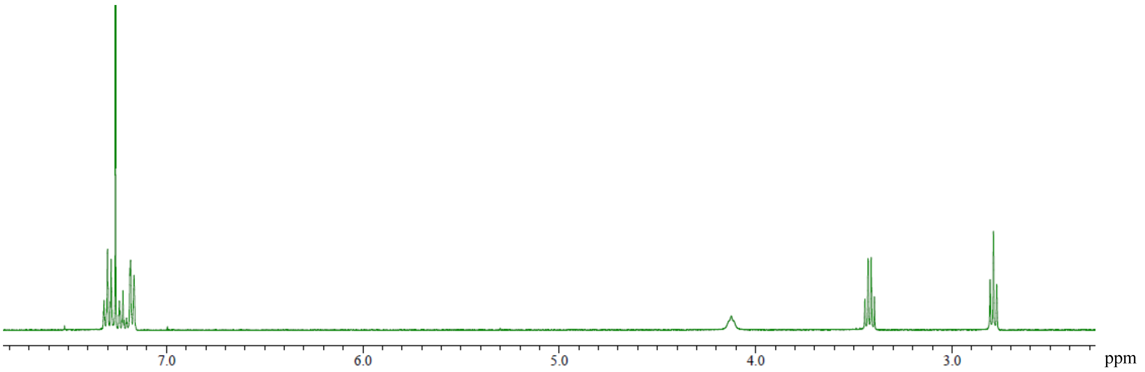

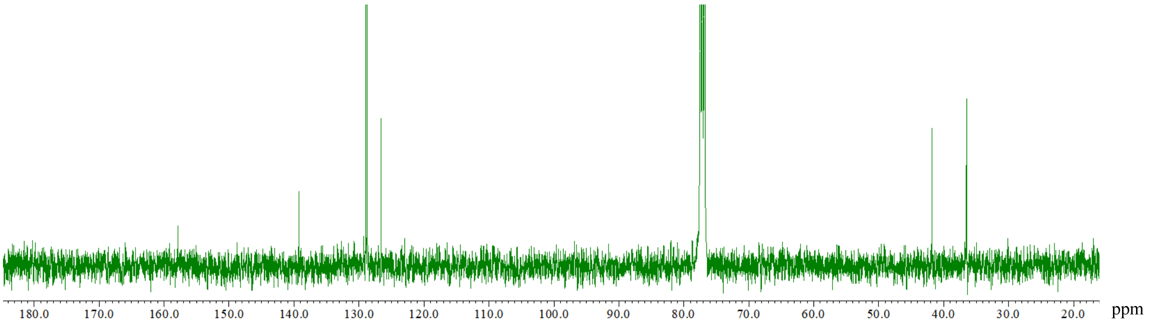

N,N'-Bis[2-(4-bromophenyl)ethyl]urea (**2b**) [CAS Registry No. 1355204-95-3]: ^{1}H NMR (400 MHz, $CDCl_3$) δ 7.42 (d, J = 8.2 Hz, 4H), 7.05 (d, J = 8.2 Hz, 4H), 4.11 (br, 2H), 3.42-3.37 (m, 4H), 2.75 (t, J = 6.8 Hz, 4H); ^{13}C NMR (100 MHz, $CDCl_3$) 157.5, 138.1, 131.7, 130.5, 120.3, 41.4, 35.8 ppm; HRMS (FAB) *m/z* Calcd. for $C_{17}H_{19}Br_2N_2O$ ($[M + H]^+$), 424.9859; Found,424.9856.

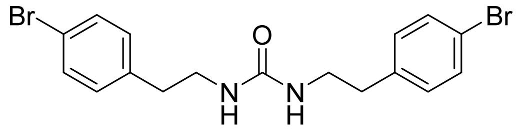

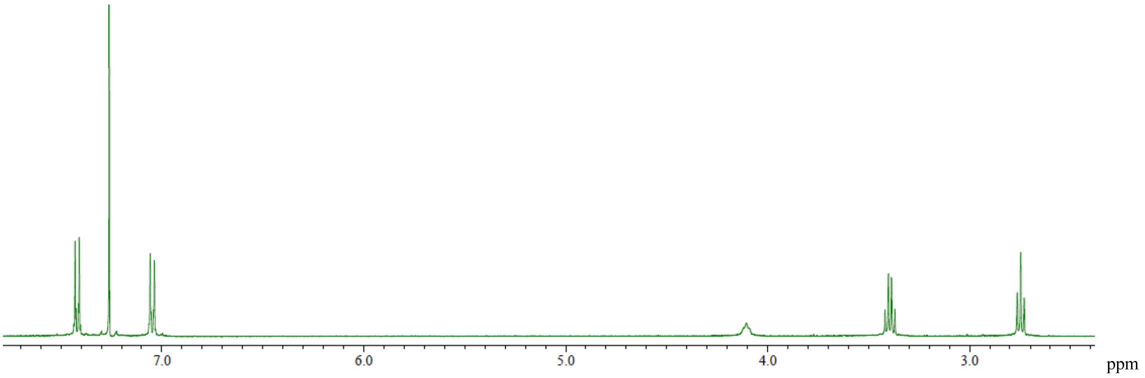

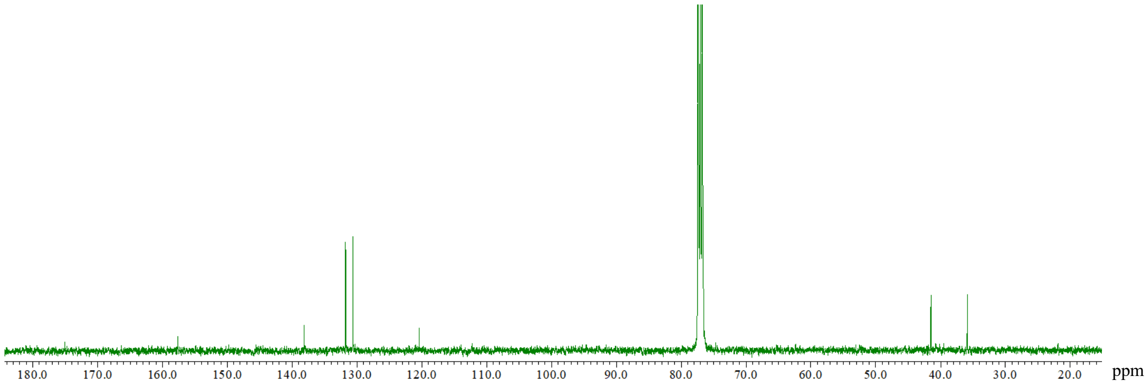

N,*N'*-Bis[2-(4-methylphenyl)ethyl)urea (**2c**) [CAS Registry No. 1900254-27-4]: ^{1}H NMR (400 MHz, $CDCl_3$) *δ* 7.10-7.7.03 (m, 8H), 4.21 (br, 2H), 3.38-3.34 (m, 4H), 2.72 (t, *J* = 6.9 Hz, 4H), 2.31 (s, 6H); ^{13}C NMR (100 MHz, $CDCl_3$) 158.2, 136.2, 135.8, 129.4, 128.8, 42.0, 35.8, 21.1 ppm; HRMS (ESI) *m/z* Calcd for $C_{19}H_{24}N_2ONa$ ($[M + Na]^+$), 319.1786; Found, 319.1806.

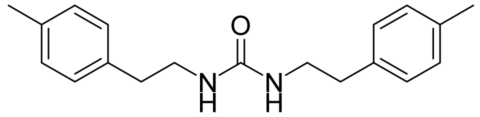

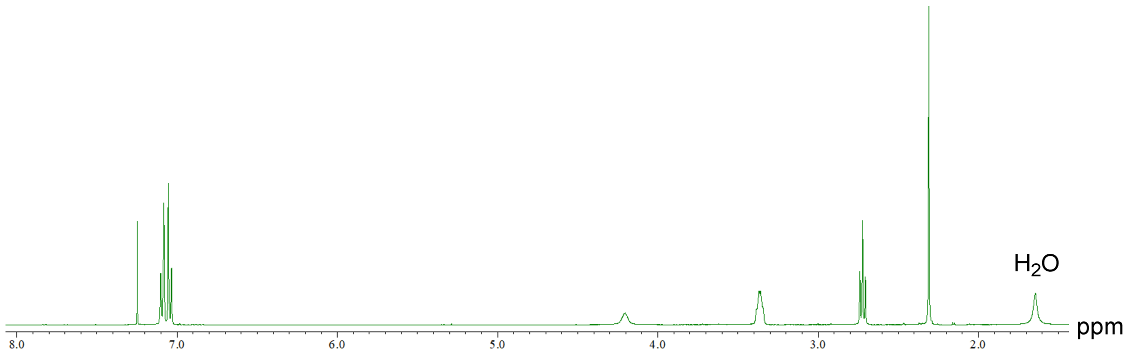

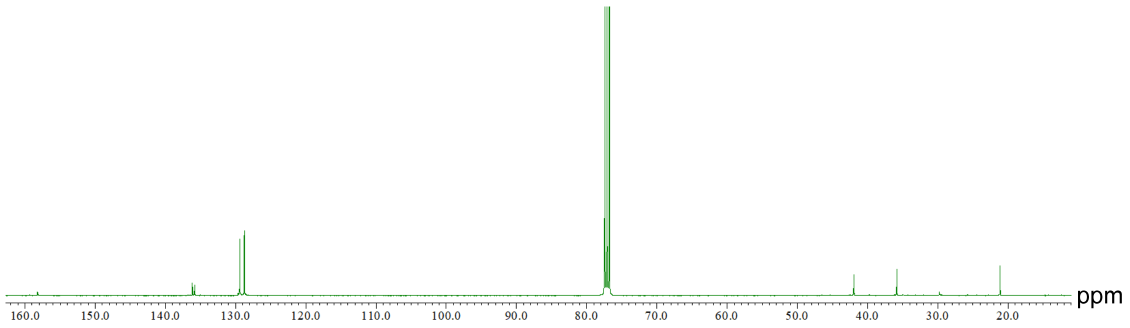

N,*N'*-Bis(2-Phenylpropyl)urea (**2d**) [CAS Registry No. 1007781-77-2]: ^{1}H NMR (400 MHz, $CDCl_3$) δ 7.31-7.13 (m, 10H), 3.99 (br, 2H), 3.48-3.42 (m, 2H), 3.13-3.05 (m, 2H), 2.92-2.81 (m, 2H), 1.22 (d, J = 6.9 Hz, 6H); ^{13}C NMR (100 MHz, $CDCl_3$) 158.1, 144.2, 128.8, 127.3, 126.8, 47.4, 40.2, 19.3 ppm; HRMS (ESI) *m/z* Calcd for $C_{19}H_{24}N_2O_1Na$ ($[M + Na]^+$), 319.1786; Found, 319.1797.

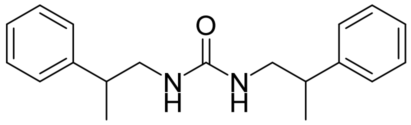

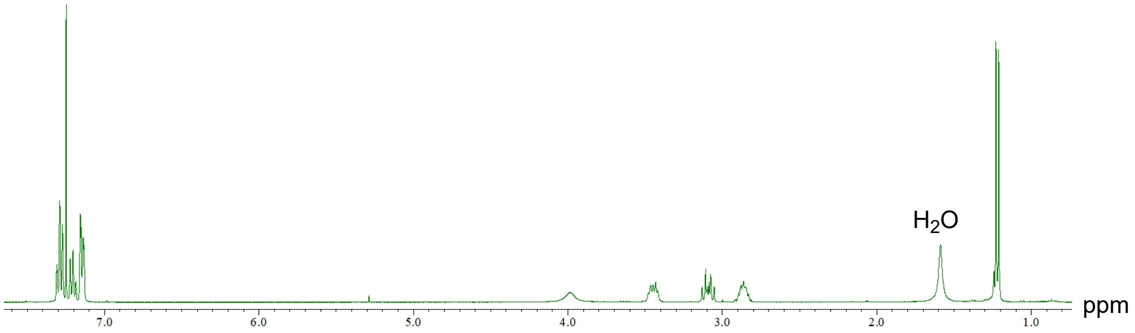

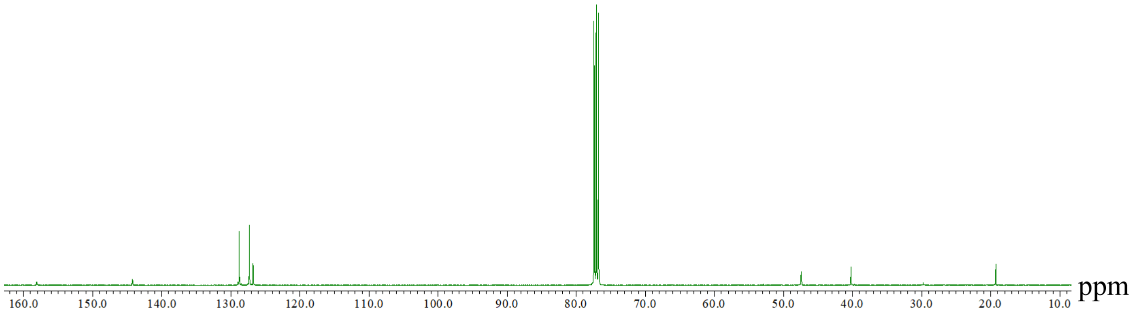

N,*N'*-Bis(3-phenylpropyl)urea (**2e**) [CAS Registry No. 1809527-04-5]: ^{1}H NMR (400 MHz, $CDCl_3$) δ 7.28-7.15 (m, 10H), 4.23 (br, 2H), 3.18-3.12 (m, 4H), 2.63 (t, J = 7.6 Hz, 4H), 1.80 (quint, J = 7.6 Hz, 4H); ^{13}C NMR (100 MHz, $CDCl_3$) 158.4, 141.6, 128.6, 128.5, 126.1, 40.3, 33.3, 31.8 ppm; HRMS (ESI) *m/z* Calcd for $C_{19}H_{24}N_2ONa$ ([M + Na]$^+$), 319.1786; Found, 319.1801.

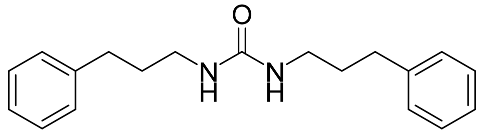

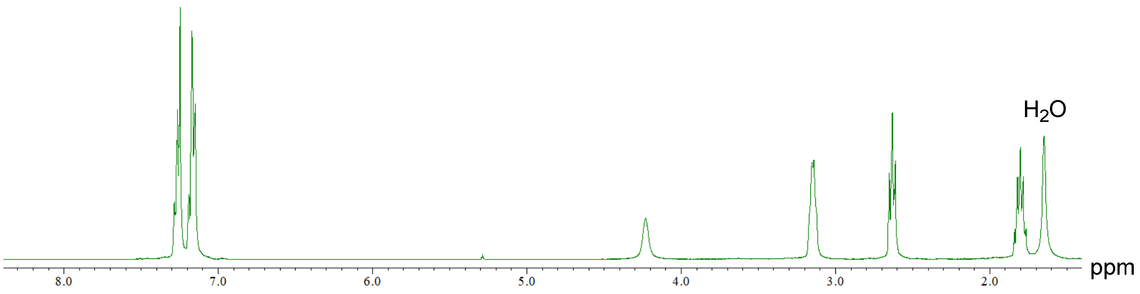

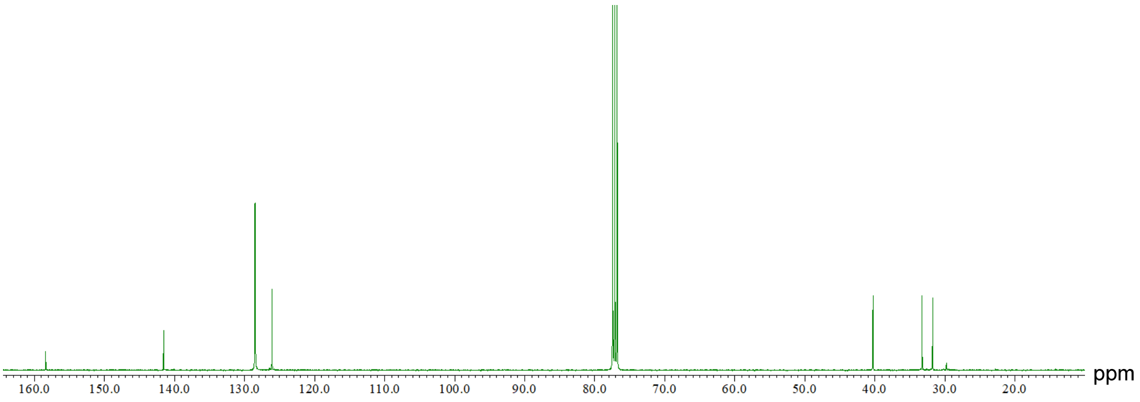

N,N'-Bis(3,3-diphenylpropyl)urea (**2f**) [CAS Registry No. 100938-83-8]: ^{1}H NMR (400 MHz, $CDCl_3$) δ 7.34-7.22 (m, 10H), 5.32 (br, 2H), 3.94 (t, J = 7.8 Hz, 2H), 3.25-3.20 (m, 4H), 2.27 (q, J = 7.4 Hz, 4H); ^{13}C NMR (100 MHz, $CDCl_3$) 170.5, 144.3, 128.8, 127.9, 126.6, 49.4, 38.9, 35.2 ppm; HRMS (ESI) *m/z* Calcd for $C_{31}H_{32}N_2ONa$ ([M + Na]$^+$), 471.2412; Found, 471.2406.

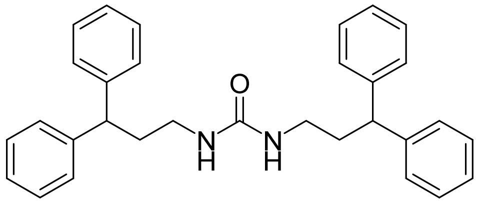

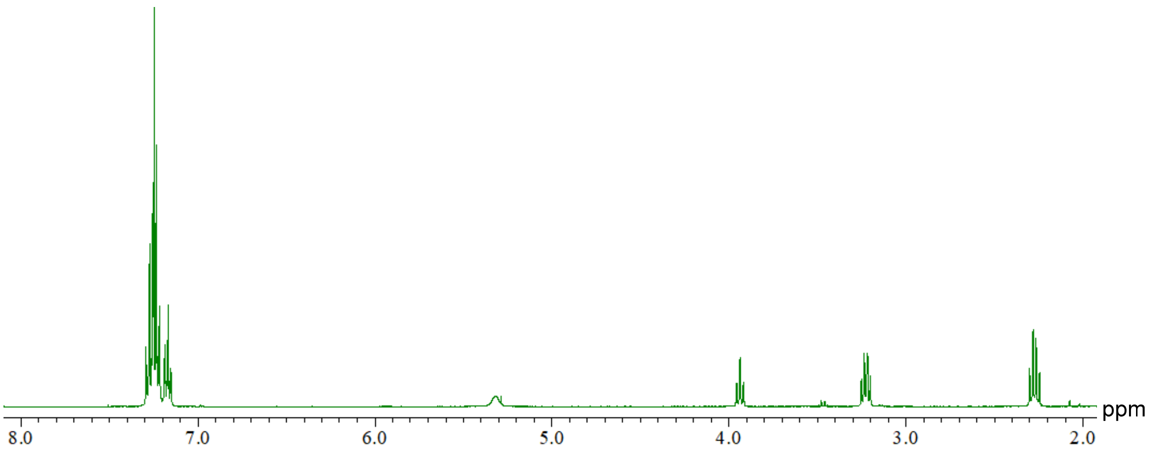

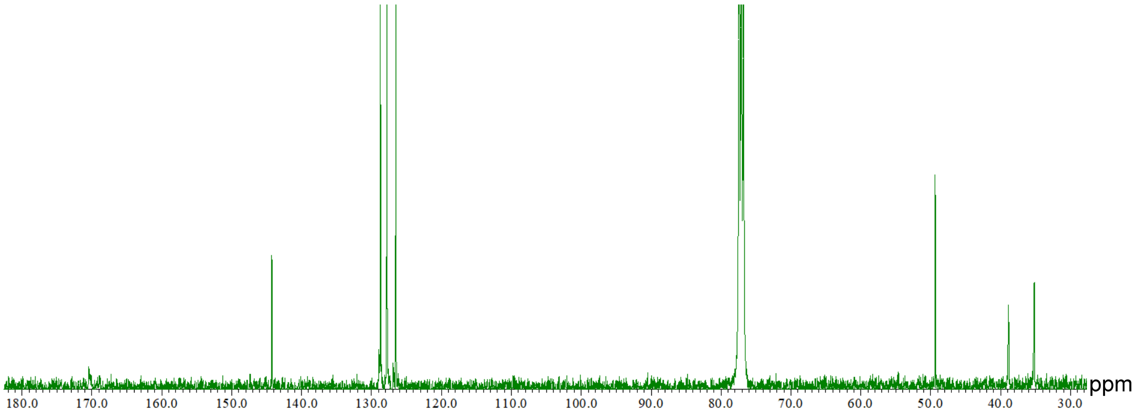

N,N'-Bis(phenylmethyl)urea (**2g**) [CAS Registry No. 1466-67-7]: ^{1}H NMR (400 MHz, $CDCl_3$) δ 7.34-7.22 (m, 10H), 4.75 (br, 2H), 4.35 (d, J = 6.0 Hz, 4H); ^{13}C NMR (100 MHz, $CDCl_3$) 158.1, 139.2, 128.8, 127.6, 127.5, 44.8 ppm; HRMS (EI) *m/z* Calcd for $C_{15}H_{16}N_2O$ ($[M]^+$), 240.1263; Found, 240.1264.

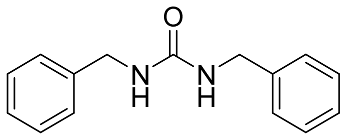

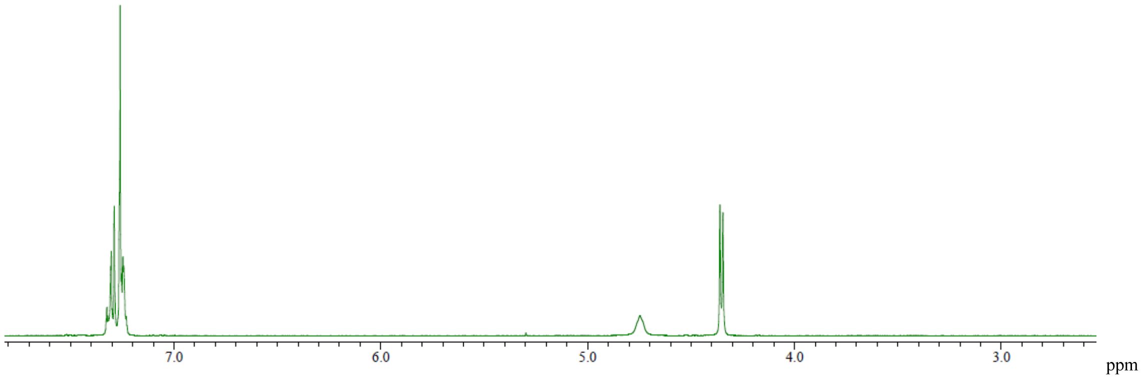

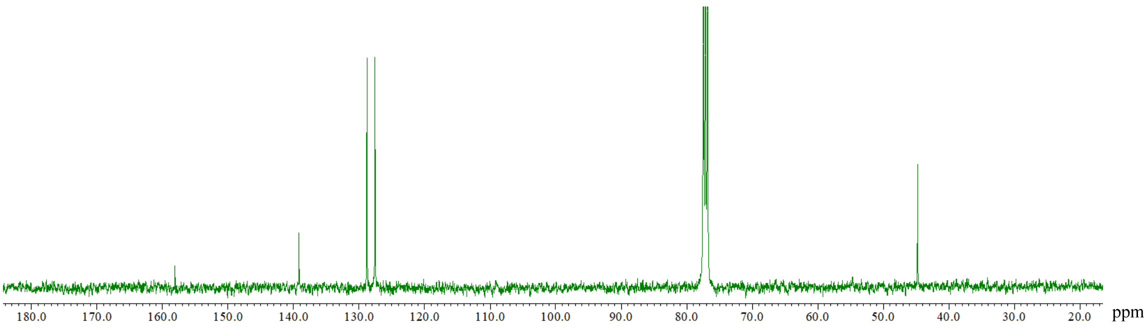

N,N′-Bis[(4-trifluoromethylphenyl)methyl]urea (**2h**) [CAS Registry No. 853180-18-4]: ^{1}H NMR (400 MHz, DMSO-*d*$_6$) δ 7.64 (d, J = 8.0 Hz, 4H), 7.42 (d, J = 8.0 Hz, 4H), 6.71 (t, J = 6.0 Hz, 2H), 4.27 (d, J = 6.0 Hz, 4H); ^{13}C NMR (100 MHz, DMSO-*d*$_6$) 158.6, 146.6, 128.1, 127.7 (q, $^{2}J_{\text{F-C}}$ = 31.6 Hz), 125.6 (q, $^{3}J_{\text{F-C}}$= 3.5 Hz), 124.9 (q, $^{1}J_{\text{F-C}}$ = 272.2 Hz), 43.1;^{19}F NMR (377 Hz, DMSO-*d*$_6$) -61.6 ppm; HRMS (ESI) *m/z* Calcd for $C_{17}H_{14}F_6N_2ONa$ ([M + Na]$^+$), 399.0908; Found, 399.0914.

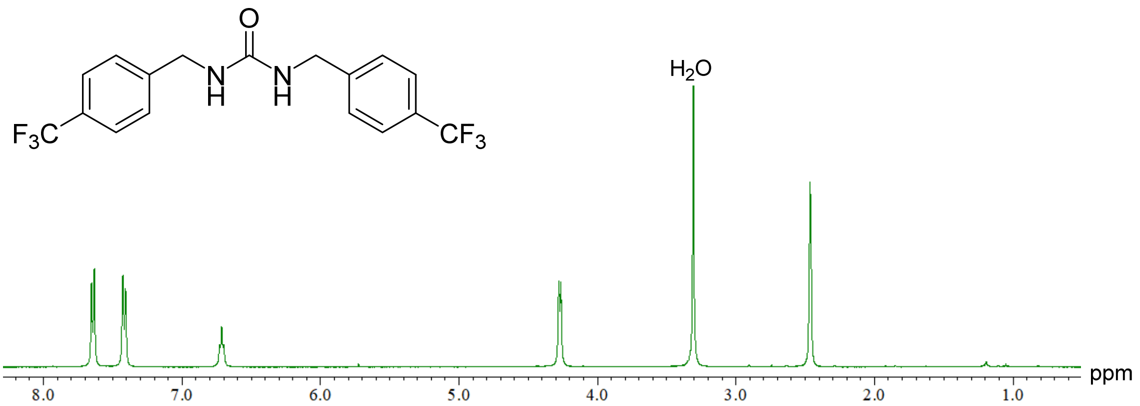

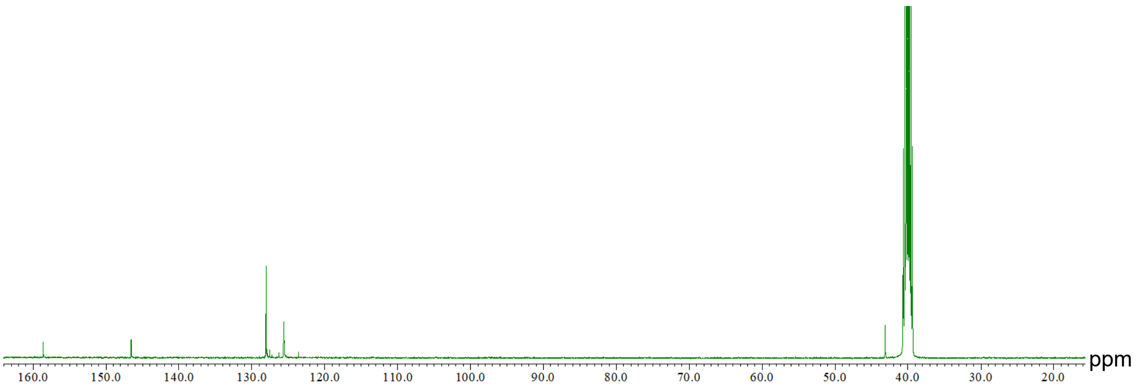

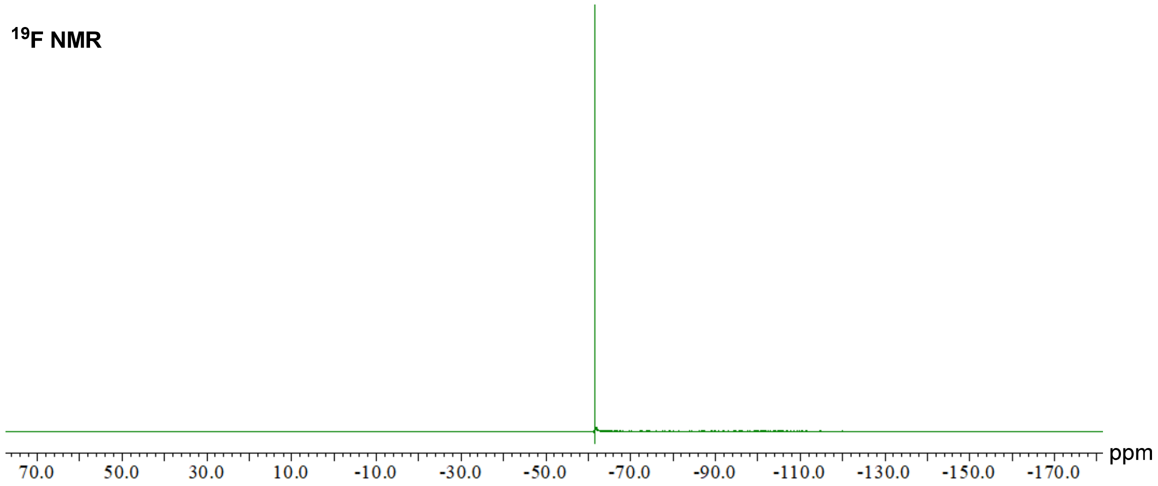

N,N'-Bis[(1*S*)-1-phenylethyl]urea (**2i**) [CAS Registry No. 19035-04-2]: ^{1}H NMR (400 MHz, DMSO-*d*$_6$) δ 7.33-7.19 (m, 10H), 6.26 (d, *J* = 8.4 Hz, 2H), 4.74-4.67 (m, 2H), 1.28 (d, *J* = 7.2 Hz, 6H); ^{13}C NMR (100 MHz, DMSO-*d*$_6$) 156.5, 145.8, 128.2, 126.5, 125.8, 48.6, 23.4 ppm; HRMS (ESI) *m/z* Calcd for $C_{17}H_{20}N_2NaO_1$ ([M + Na]$^+$), 291.14733; Found, 291.14677.

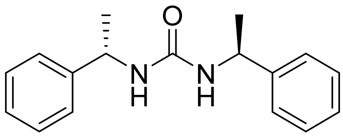

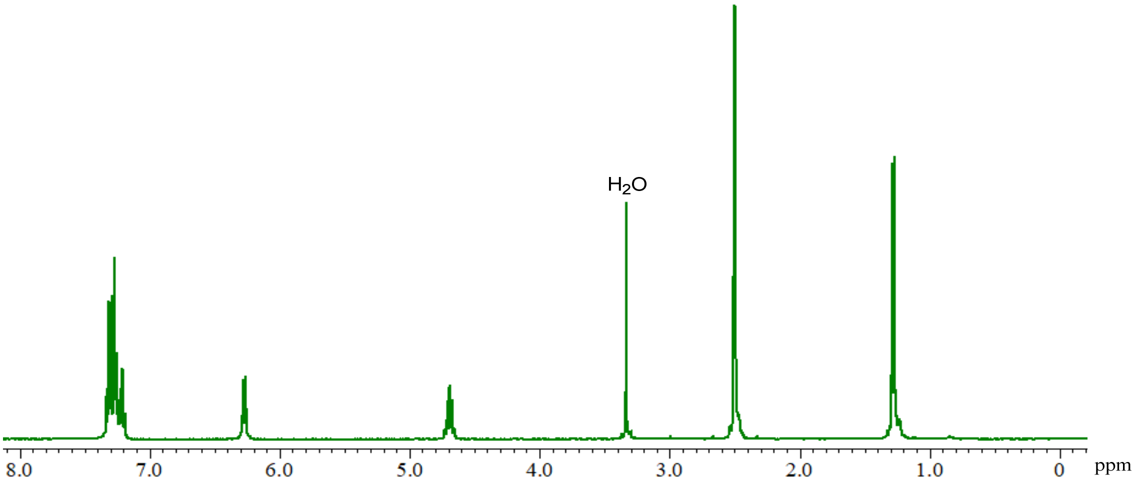

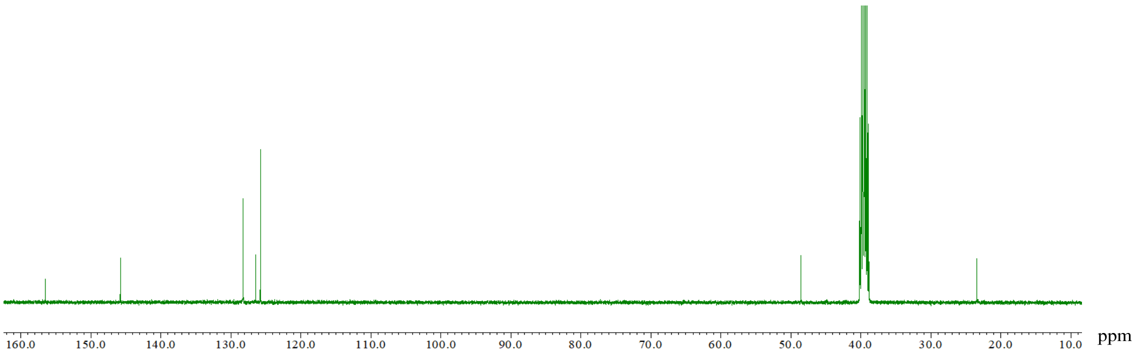

N,N'-Dihexylurea (**2j**) [CAS Registry No. 2763-88-4]: ^{1}H NMR (400 MHz, $CDCl_3$) δ 4.30 (br, 2H), 3.18-3.14 (m, 4H), 1.54-1.47 (m, 4H), 1.34-1.25 (m, 12H), 0.89 (t, J = 6.8 Hz, 6H); ^{13}C NMR (100 MHz, $CDCl_3$) 158.3, 41.0, 31.7, 30.3, 26.7, 22.7, 14.2 ppm; HRMS (EI) *m/z* Calcd. for $C_{13}H_{28}N_2O$ ($[M]^+$), 228.2202; Found, 228.2204.

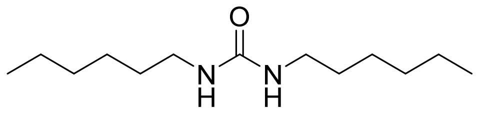

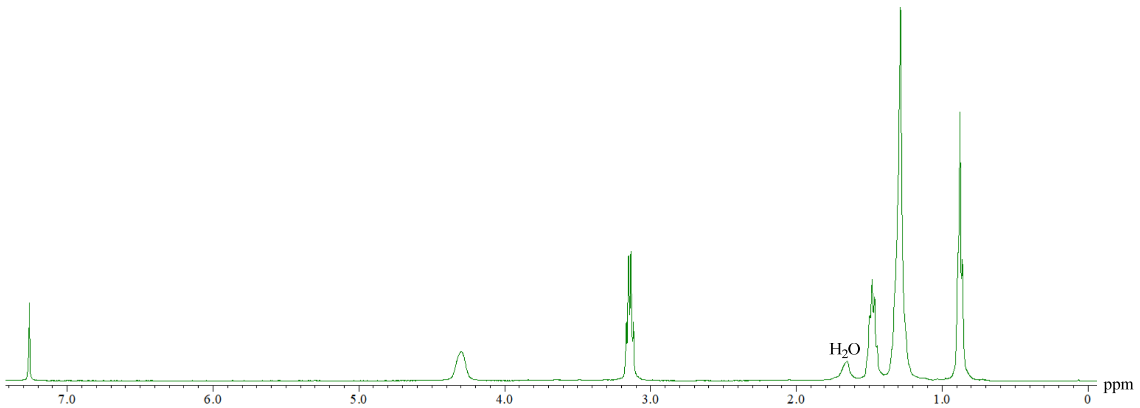

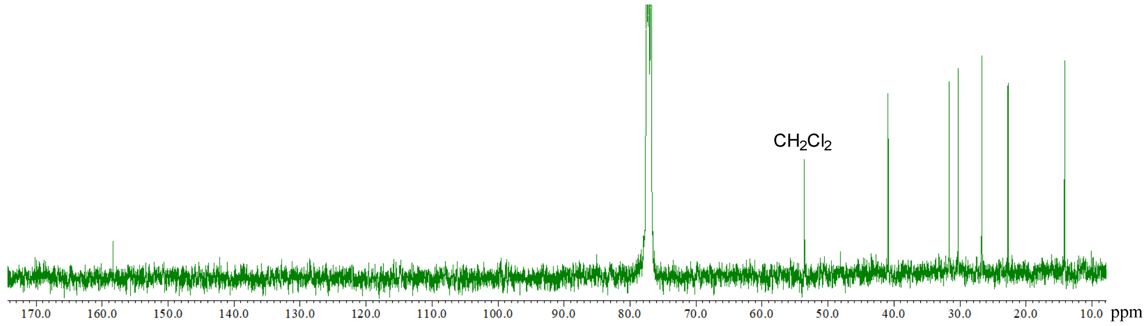

N,N'-Didecylurea (**2k**) [CAS Registry No. 1943-09-5]: ^{1}H NMR (400 MHz, CD_3OD) δ 3.06 (t, J = 6.9 Hz, 4H), 1.45-1.42 (m, 4H), 1.29-1.27 (m, 28H), 0.87 (t, J = 6.9 Hz, 4H); ^{13}C NMR (100 MHz, $CDCl_3$) 158.3, 40.8, 32.0, 29.7, 29.44, 29.41, 27.0, 22.8, 14.2 ppm; HRMS (ESI) m/z Calcd for $C_{21}H_{44}N_2ONa$ ($[M + Na]^+$), 363.3351; Found, 363.3356.

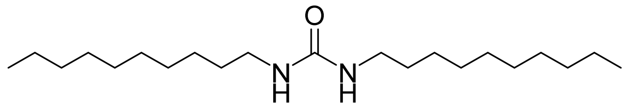

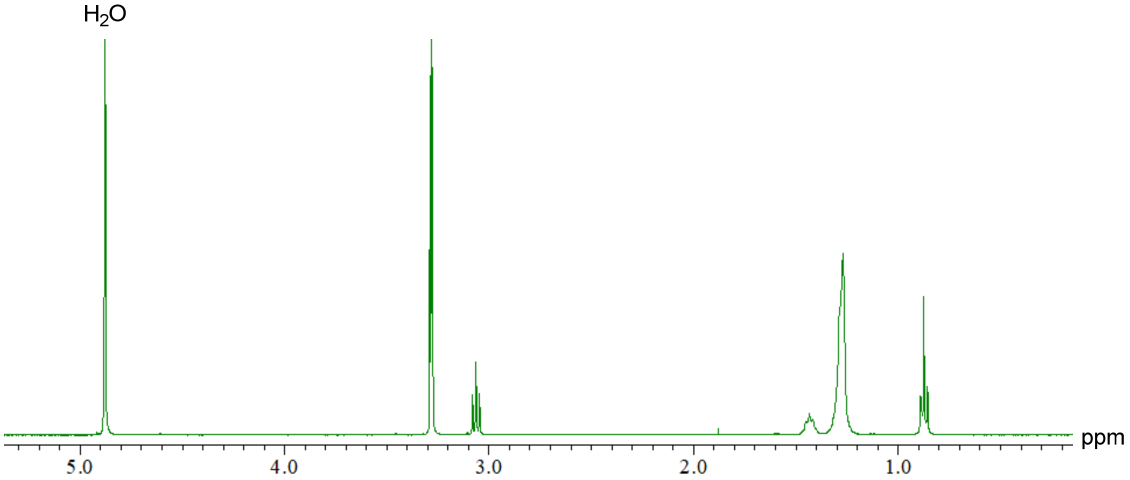

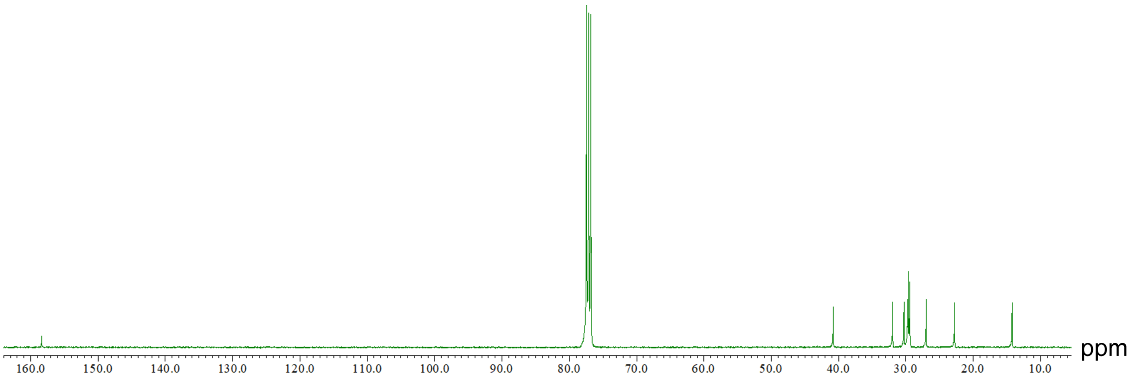

N,N′-Bis(3-ethoxypropyl)urea (**2l**) [CAS Registry No. 2205524-12-3]: ^{1}H NMR (400 MHz, $CDCl_3$) δ 5.23 (br, 2H), 3.51-3.44 (m, 8H), 3.27 (t, J = 6.3 Hz, 4H), 1.80-1.1.74 (m, 4H), 1.19 (t, J = 7.0 Hz, 6H); ^{13}C NMR (100 MHz, $CDCl_3$) 158.8, 69.0, 66.5, 39.1, 29.8, 15.3 ppm; HRMS (ESI) *m/z* Calcd for $C_{11}H_{24}N_2O_3Na$ ($[M + Na]^+$), 255.1685; Found, 255.1683.

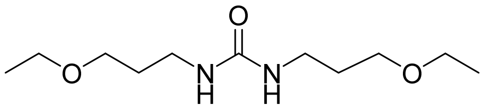

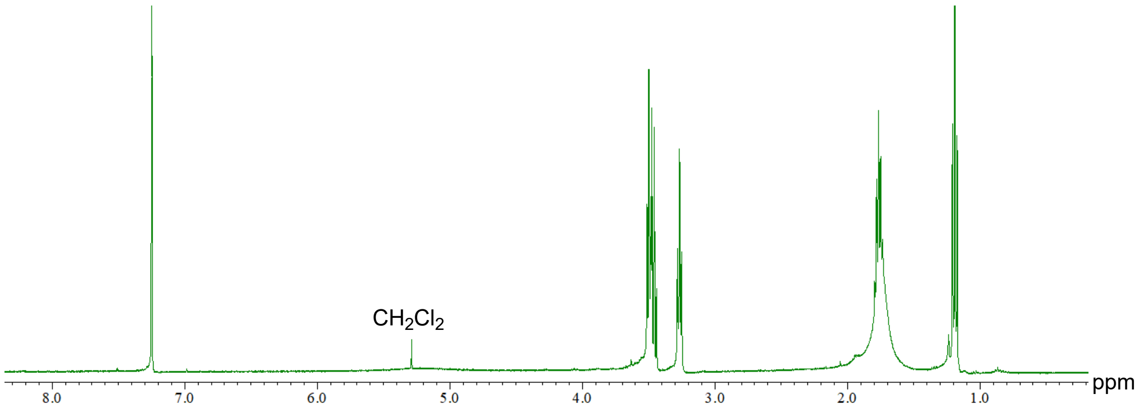

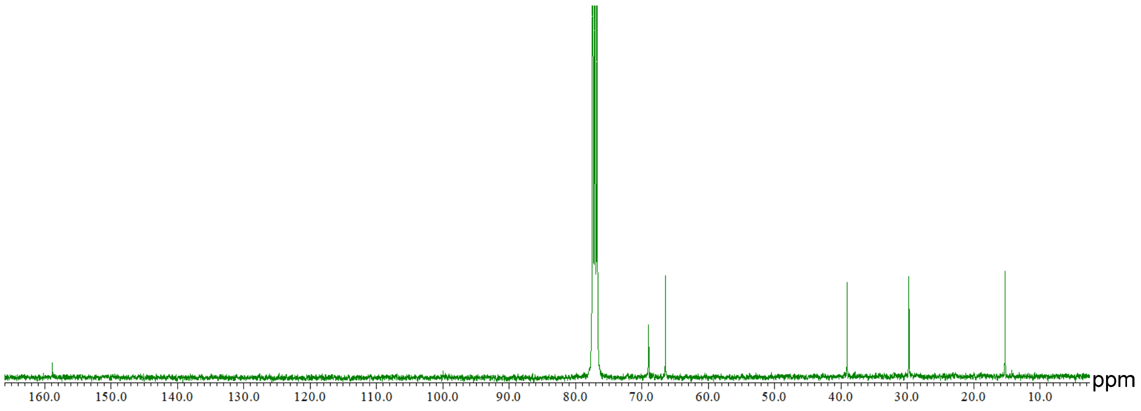

N,N′-Bis[(tetrahydrofuran-2-yl)methyl]urea (**2m**) [CAS Registry No. 875923-29-8]: ^{1}H NMR (400 MHz, $CDCl_3$) δ 5.12 (br, 2H), 3.98-3.92 (m, 2H), 3.87-3.81 (m, 2H), 3.76-3.70 (m, 2H), 3.47-3.43 (m, 2H), 3.11-3.06 (m, 2H), 1.98-1.81 (m, 6H), 1.61-1.53 (m, 2H); ^{13}C NMR (100 MHz, CD_3CN) 159.5, 79.2, 68.5, 44.7, 29.2, 26.5 ppm; HRMS (ESI) *m/z* Calcd for $C_{11}H_{20}N_2O_3Na$ ($[M + Na]^+$), 251.1371; Found, 251.1376.

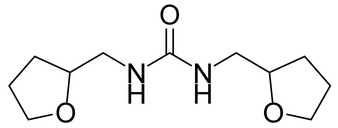

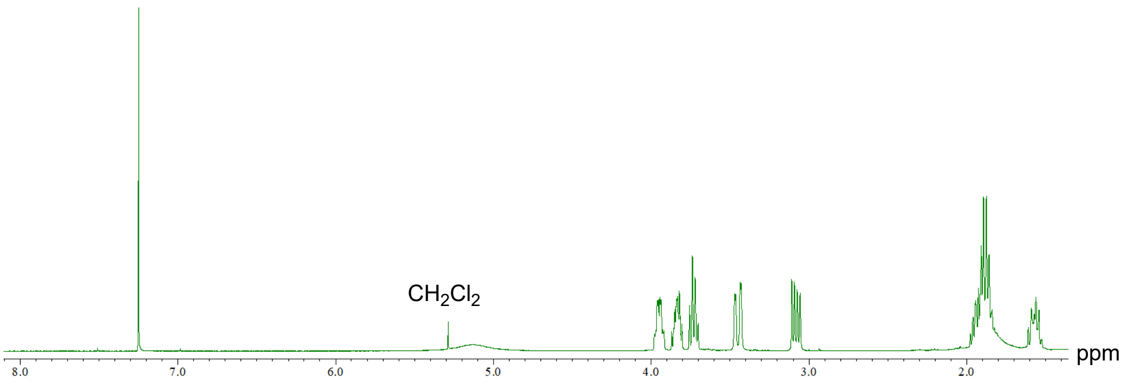

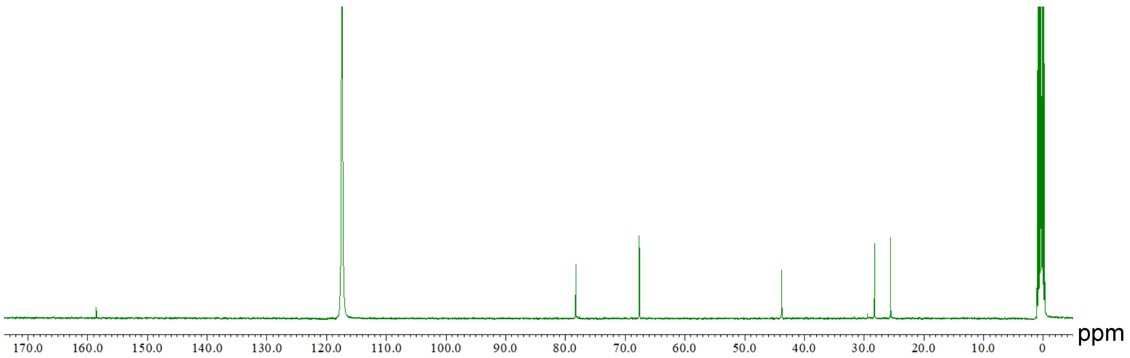

N,N′-Dicyclopentylurea (**2n**) [CAS Registry No. 58713-33-0]: ^{1}H NMR (400 MHz, $CDCl_3$) δ 4.26 (br, 2H), 3.97-3.90 (m, 2H), 1.99-1.91 (m, 4H), 1.70-1.54 (m, 8H), 1.40-1.33 (m, 4H); ^{13}C NMR (100 MHz, $CDCl_3$) 157.9, 52.4, 33.8, 23.7 ppm; HRMS (ESI) *m/z* Calcd for $C_{11}H_{20}N_2ONa$ ([M + Na]$^+$), 219.1473; Found, 219.1461.

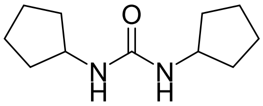

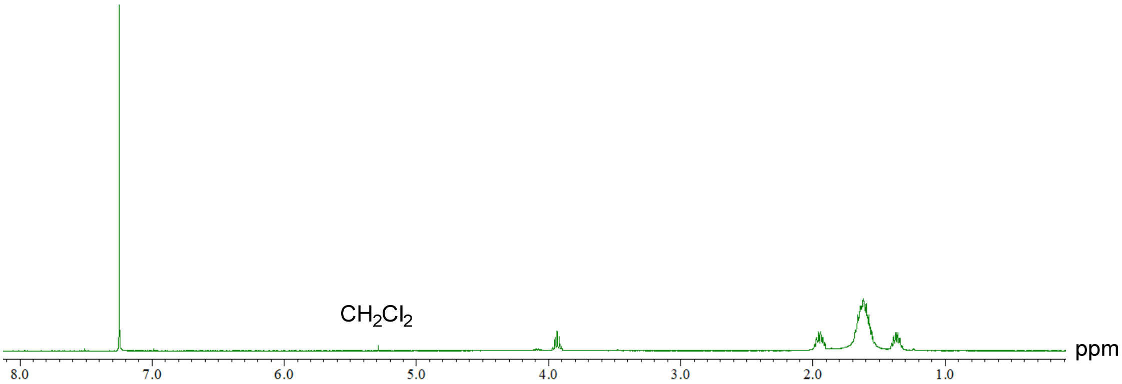

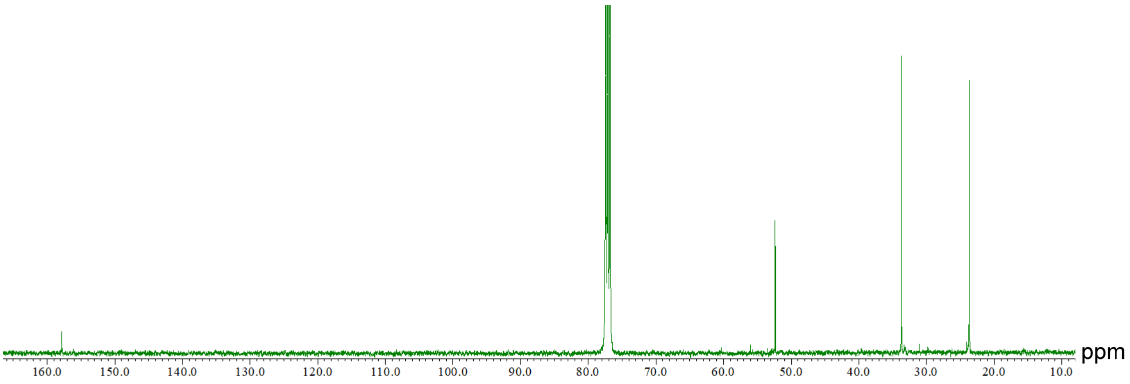

N,N'-Dicyclohexylurea (**2o**) [CAS Registry No. 2387-23-7]: ^{1}H NMR (400 MHz, $CDCl_3$) δ 4.12 (br, 2H), 3.54-3.42 (m, 2H), 2.00-1.88 (m, 4H), 1.78-1.50 (m, 6H), 1.42-1.25 (m, 4H), 1.25-1.03 (m, 6H); ^{13}C NMR (100 MHz, $CDCl_3$) 156.9, 49.4, 34.1, 25.8, 25.1 ppm; HRMS (EI) *m/z* Calcd for $C_{13}H_{24}N_2O$ ($[M]^+$), 224.1889; Found, 224.1887.

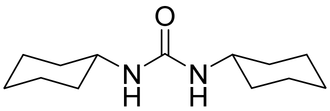

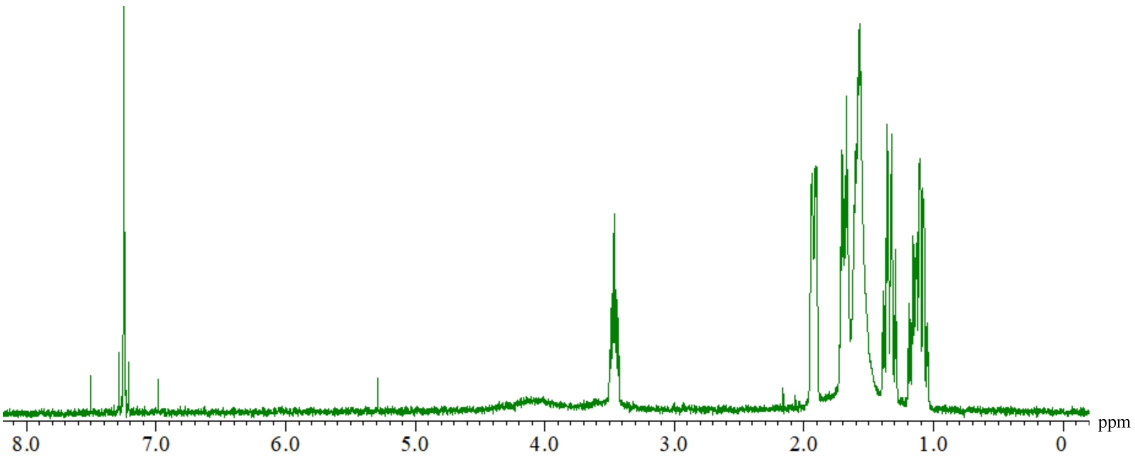

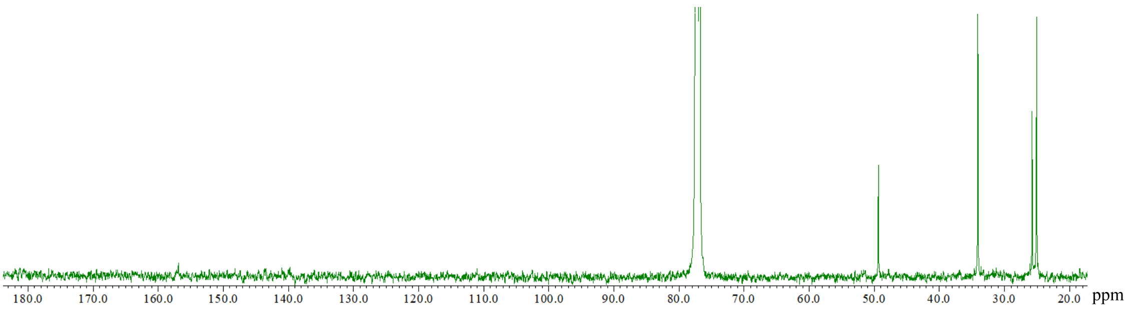

N,N'-Bis(tricyclo[3.3.1.1^{3,7}]dec-1-yl)urea (**2p**) [CAS Registry No. 29559-44-2]: ^{1}H NMR (400 MHz, $CDCl_3$) *δ* 3.82 (br, 2H), 2.05 (br, 6H), 1.96-1.92 (m, 12H), 1.68-1.63 (m, 12H); ^{13}C NMR (100 MHz, $CDCl_3$) 156.3, 51.2, 42.7, 36.6, 29.7 ppm; HRMS (FAB) *m/z* Calcd for $C_{21}H_{33}N_2O$ ([M + H]$^{+}$), 329.2587; Found, 329.2594.

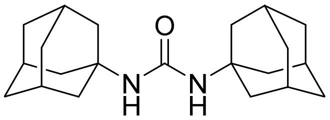

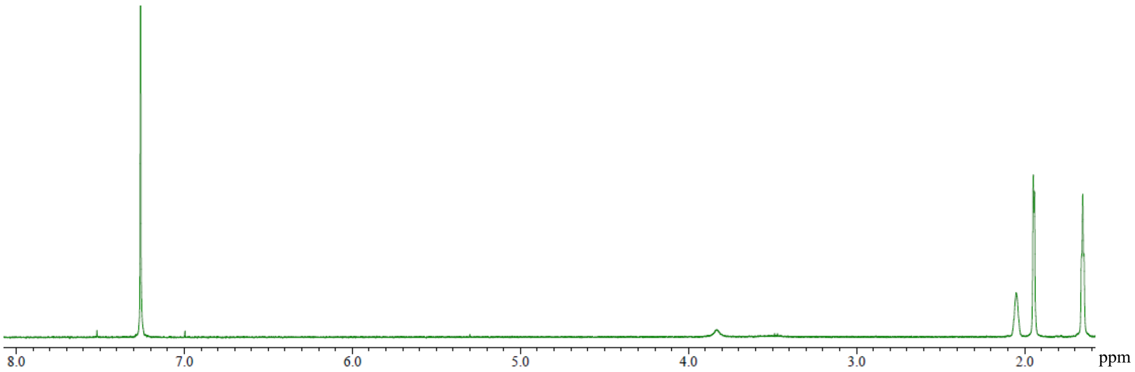

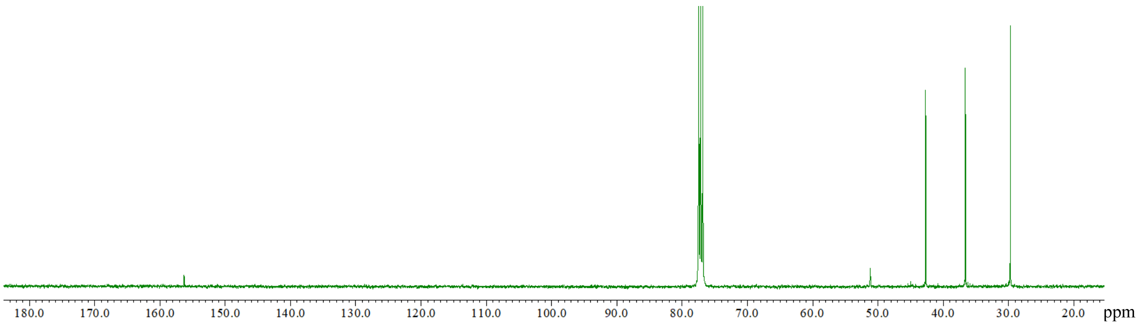

N,N'-Bis(4-methoxyphenyl)urea (**2q**) [CAS Registry No. 1227-44-7]: ^{1}H NMR (400 MHz, DMSO-d_6) δ 8.34 (br, 2H), 7.33 (d, J = 9.2 Hz, 4H), 6.85 (d, J = 9.2 Hz, 4H), 3.71 (s, 6H); ^{13}C NMR (100 MHz, DMSO-d_6) 154.3, 152.9, 132.9, 119.9, 114.0, 55.2 ppm; HRMS (EI) *m/z* Calcd for $C_{15}H_{16}N_2O_3$ ([M]$^+$), 272.1161; Found, 272.1160.

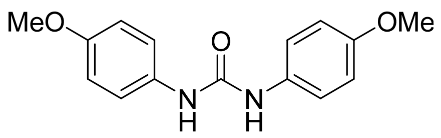

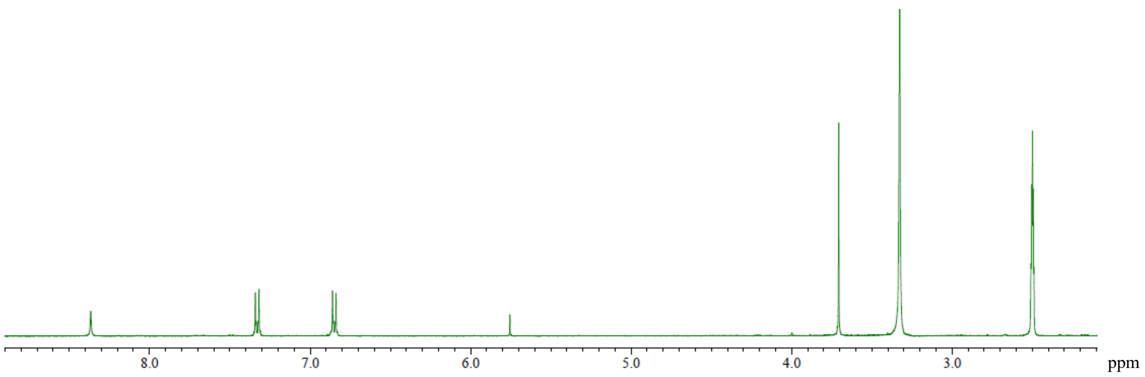

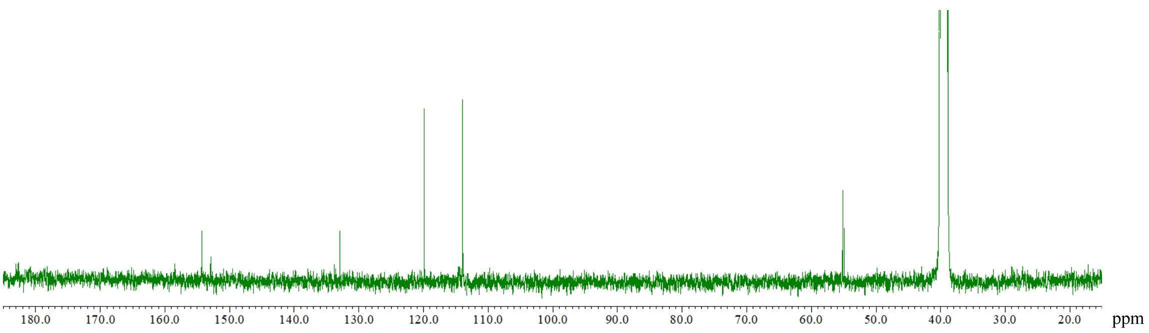

4-Phenyl-*N*-tricyclo[3.3.1.1^{3,7}]dec-1-yl-1-piperazinecarboxamide (**2ps**) [CAS Registry No. 345973-04-8]: ^{1}H NMR (400 MHz, $CDCl_3$) δ 7.28-7.30 (m, 2H), 6.87-6.94 (m, 3H), 4.24 (br 1H), 3.48 (t, J = 5.2 Hz, 4H), 3.18 (t, J = 5.2 Hz, 4H), 2.08 (br, 3H), 1.99-2.00 (m, 6H), 1.68 (br, 6H); ^{13}C NMR (100 MHz, CD_3CN) 157.5, 152.5, 130.0, 120.5, 117.1, 51.8, 49.8, 44.7, 42.8, 37.2, 30.6 ppm; HRMS (ESI) *m/z* Calcd for $C_{21}H_{29}N_3ONa$ ([M + Na]$^+$), 362.2208; Found, 362.2217.

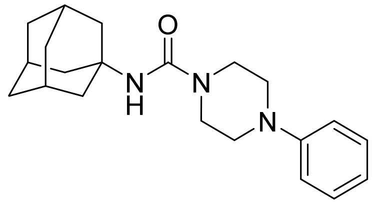

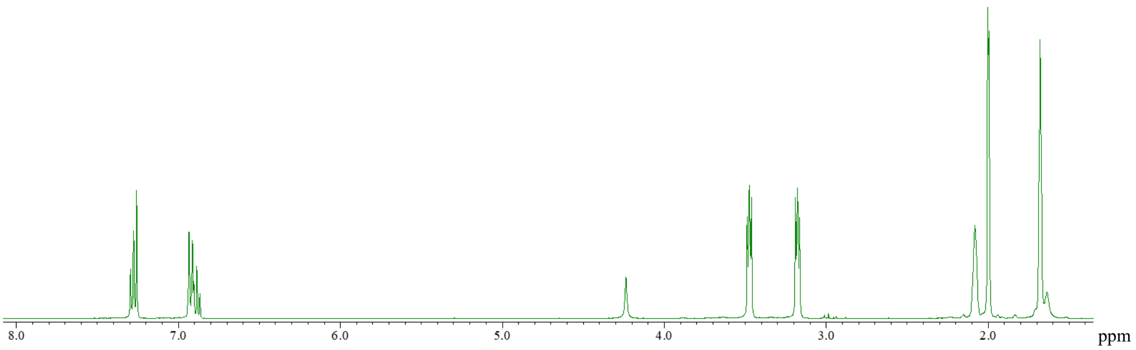

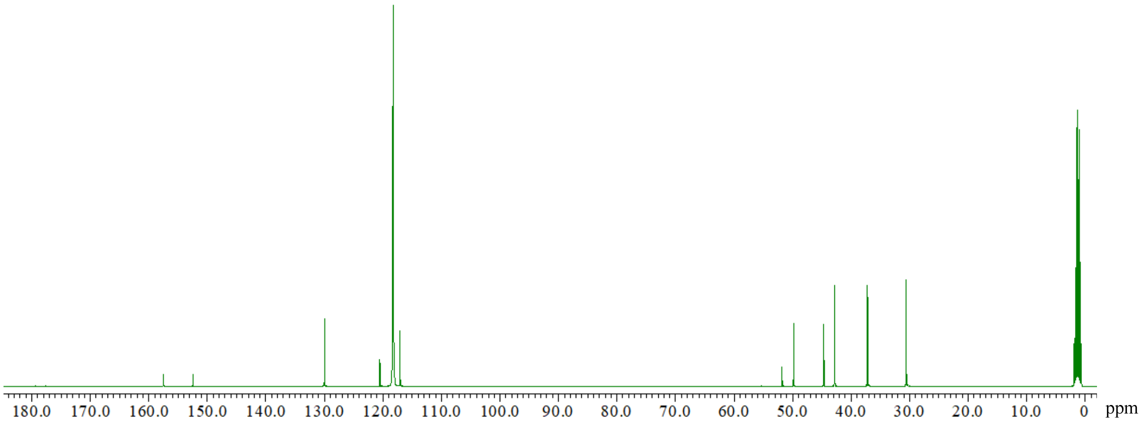

4-Phenyl-*N*-(2-phenylethyl)-1-piperidinecarboxamide (**2at**) [CAS Registry No. 1809527-27-2]: ^{1}H NMR (400 MHz, $CDCl_3$) δ 7.33-7.18 (m, 10H), 4.49 (br, 1H), 4.02-3.98 (m 2H), 3.55-3.50 (m, 2H), 2.88-2.81 (m, 4H), 2.69-2.61 (m, 1H), 1.85-1.81 (m, 2H), 1.68-1.56 (m, 2H); ^{13}C NMR (100 MHz, $CDCl_3$) 157.5, 145.5, 139.5, 128.9, 128.6, 128.5, 126.7, 126.42, 126.36, 44.6, 42.7, 42.1, 36.4, 33.0 ppm; HRMS (EI) *m/z* Calcd for $C_{20}H_{24}N_2O$ ($[M]^+$), 308.1889; Found, 308.1887.

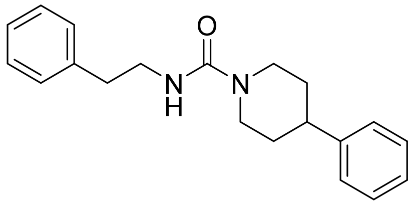

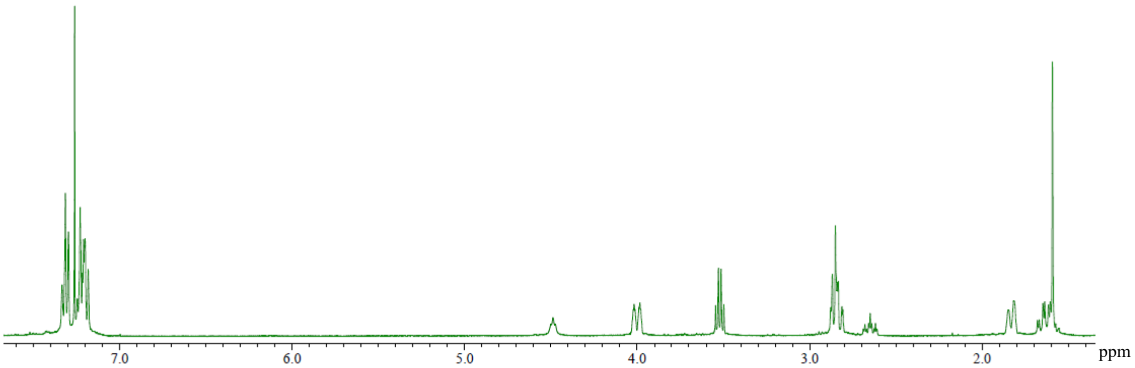

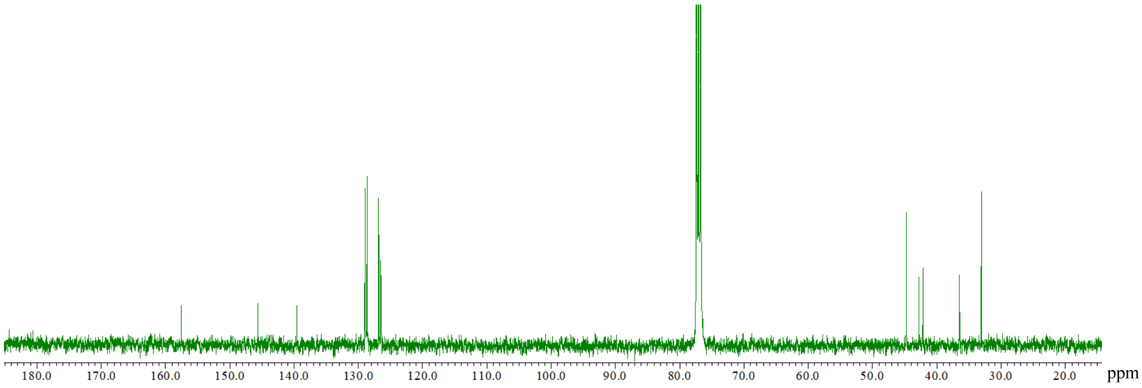

The HPLC chart for *N,N′*-bis(1-phenylethyl)urea

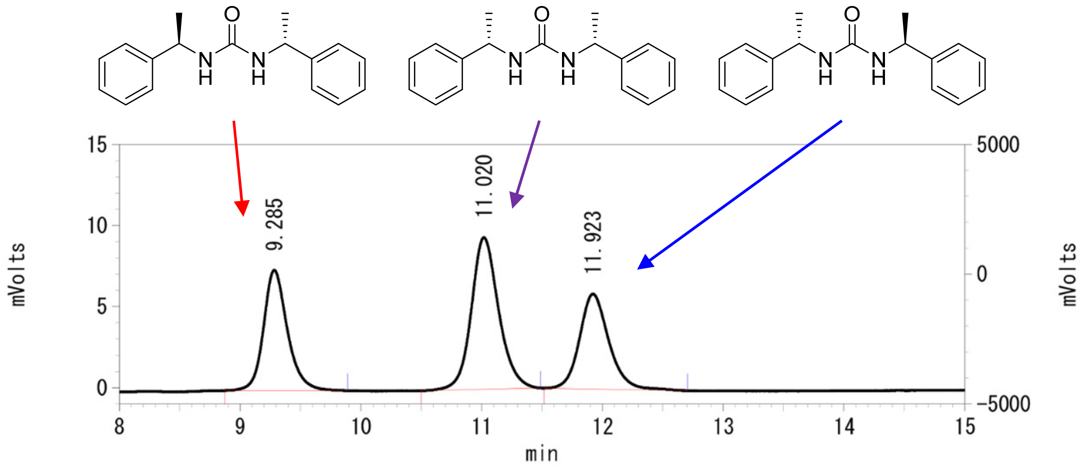

Pk #	Retention Time	Area	Area Percent
1	9.285	102434	29.318
2	11.020	147856	42.319
3	11.923	99095	28.363

The HPLC chart for *N,N′*-bis[(1*S*)-1-phenylethyl]urea (2i)

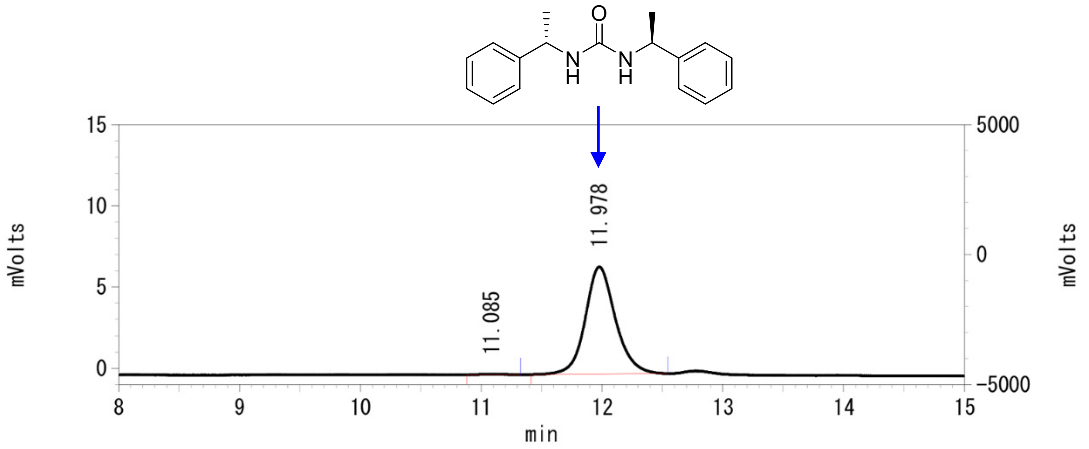

Pk #	Retention Time	Area	Area Percent
1	11.085	485	0.420
2	11.978	115106	99.580

10. References

1 D. C. Crans, H. Chen, O. P. Anderson and M. M. Miller, *J. Am. Chem. Soc.*, 1993, **115**, 6769-6776.

2 T. Moriuchi, M. Nishina and T. Hirao, *Angew. Chem. Int. Ed.*, 2010, **49**, 83-86.
